# Supplementary material for: AXL receptor tyrosine kinase regulates Golgi organization and function via an adhesion-Arf1 signalling axis in breast and lung cancer cell lines
Source: Biol Open. 2026 May 19;15(5):bio062581. doi: 10.1242/bio.062581 (PMC13225716; doi:10.1242/bio.062581)
Supplement: Supplementary information [file biolopen-15-062581-s1.pdf]

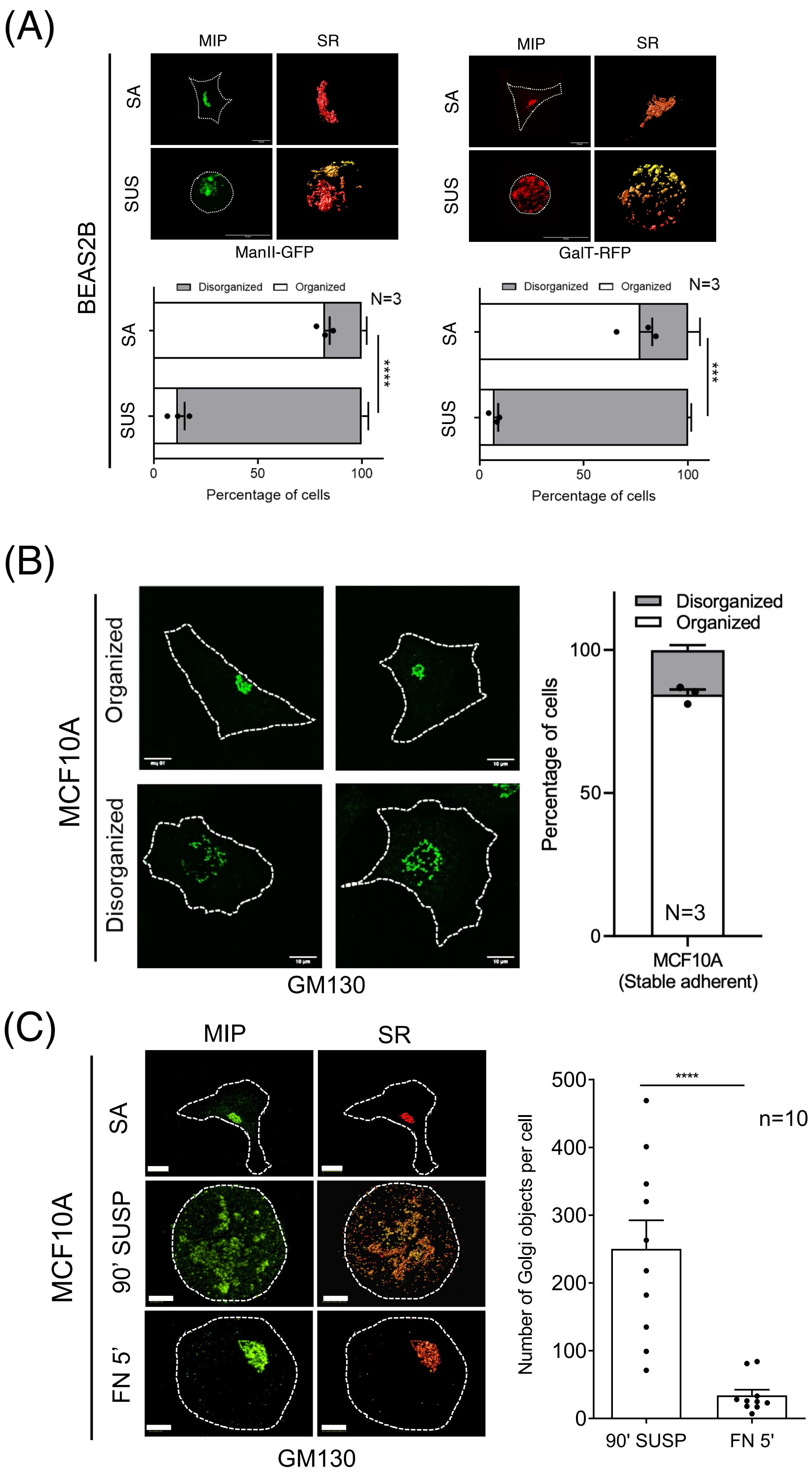

Biology Open • Supplementary information

Fig. S1. Adhesion dependent regulation of Golgi organization in non-transformed breast and lung epithelial cells. (A) BEAS2B cells transfected with ManII-GFP (green) or GalT-RFP (red). Representative deconvoluted Z-stacks images for the predominant phenotype shown as maximum intensity projection (MIP) and a zoomed image of the Golgi with surface rendering (SR). Percentage distribution profile of cells (n≥200) with organized (white) and disorganized (grey) Golgi in stable adherent (SA) and non-adherent (SUS) cells. Graphs represent mean±SEM from three independent experiments. **(B)** Stable-adherent MCF10A cells immunostained for GM130 (green). Representative cross-sectional confocal images for organized and disorganized Golgi phenotype shown. Percentage distribution profile for cells (n≥200) show organized (white) and disorganized (grey) Golgi for cells. Graph represents mean±SEM from three independent experiments.**(C)** Representative deconvoluted Z-stacks images for GM130 (green) immunostained in stable adherent (SA), non-adherent (90'SUSP) and re-adherent (FN5') MCF10A cells shown as maximum intensity projection (MIP) and surface rendered (SR) image. Graph shows mean±SEM of discontinuous cis-Golgi (GM130) objects per cell for non-adherent (90'SUSP) and re-adherent (FN5') (n=10 cells). Statistical analysis was done using one-way ANOVA multiple comparisons test with Tukey's method for error correction for the distribution profile, and Mann-Whitney U test for object count analysis. Scale bars are 10μm. (*p≤0.05, **p≤0.01, ***p≤0.001, ****p≤0.0001, ns=not significant).

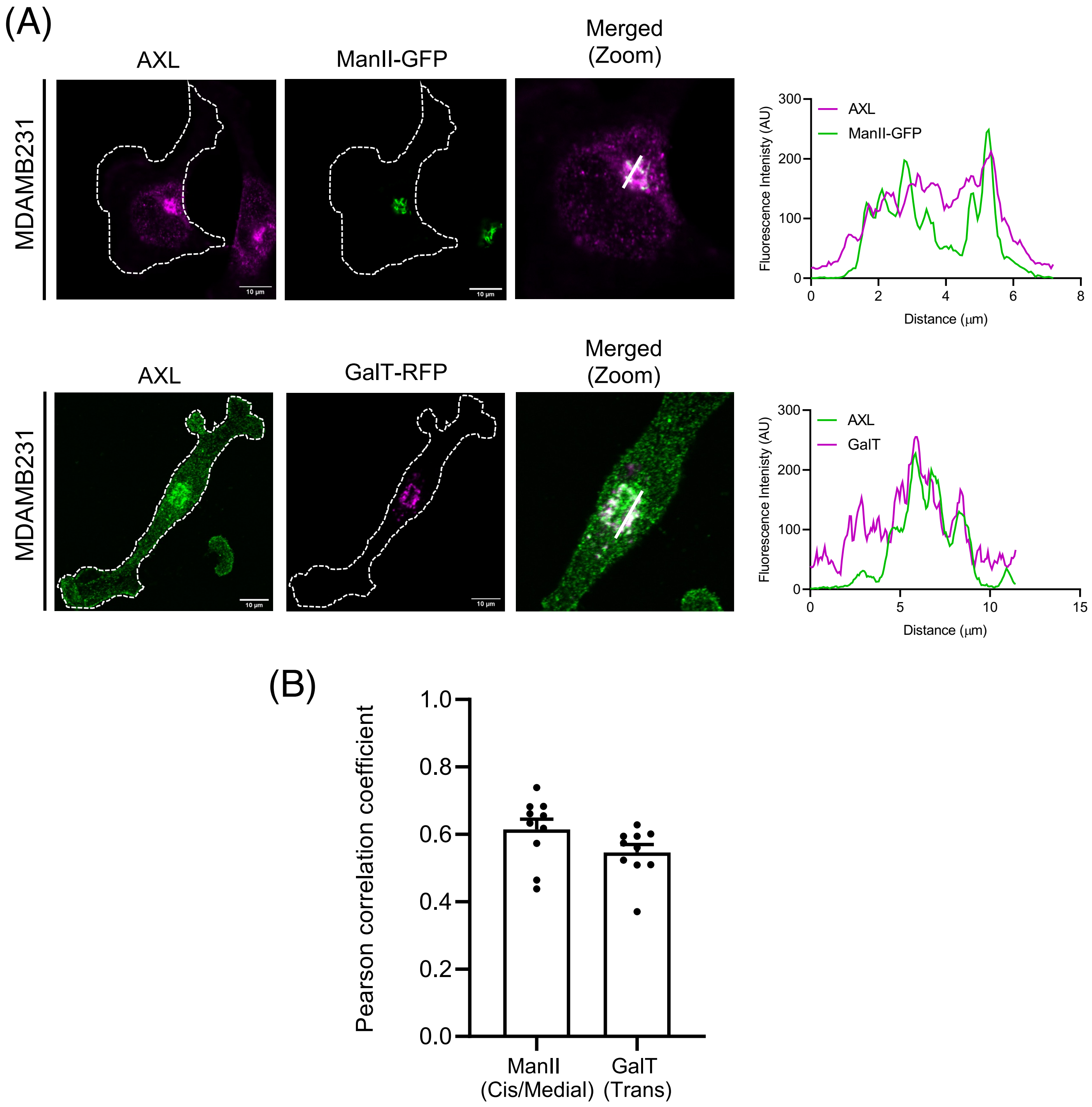

Fig. S2. AXL localization to Golgi in adherent MDAMB231 cells. (A) Representative cross-section images with merged zoomed insets of cells immunostained for endogenous AXL, expressing ManII-GFP (green) and GalT-RFP (magenta) in adherent MDAMB231 cells and corresponding line plots for AXL and Golgi marker (ManII-GFP and GalT-RFP) shown next to their respective images. **(B)** Graph represents the Pearson's correlation coefficients for AXL and Golgi marker (ManII and GalT) colocalization plotted as mean $\pm$ SEM for n=10 cells.

Biology Open • Supplementary information

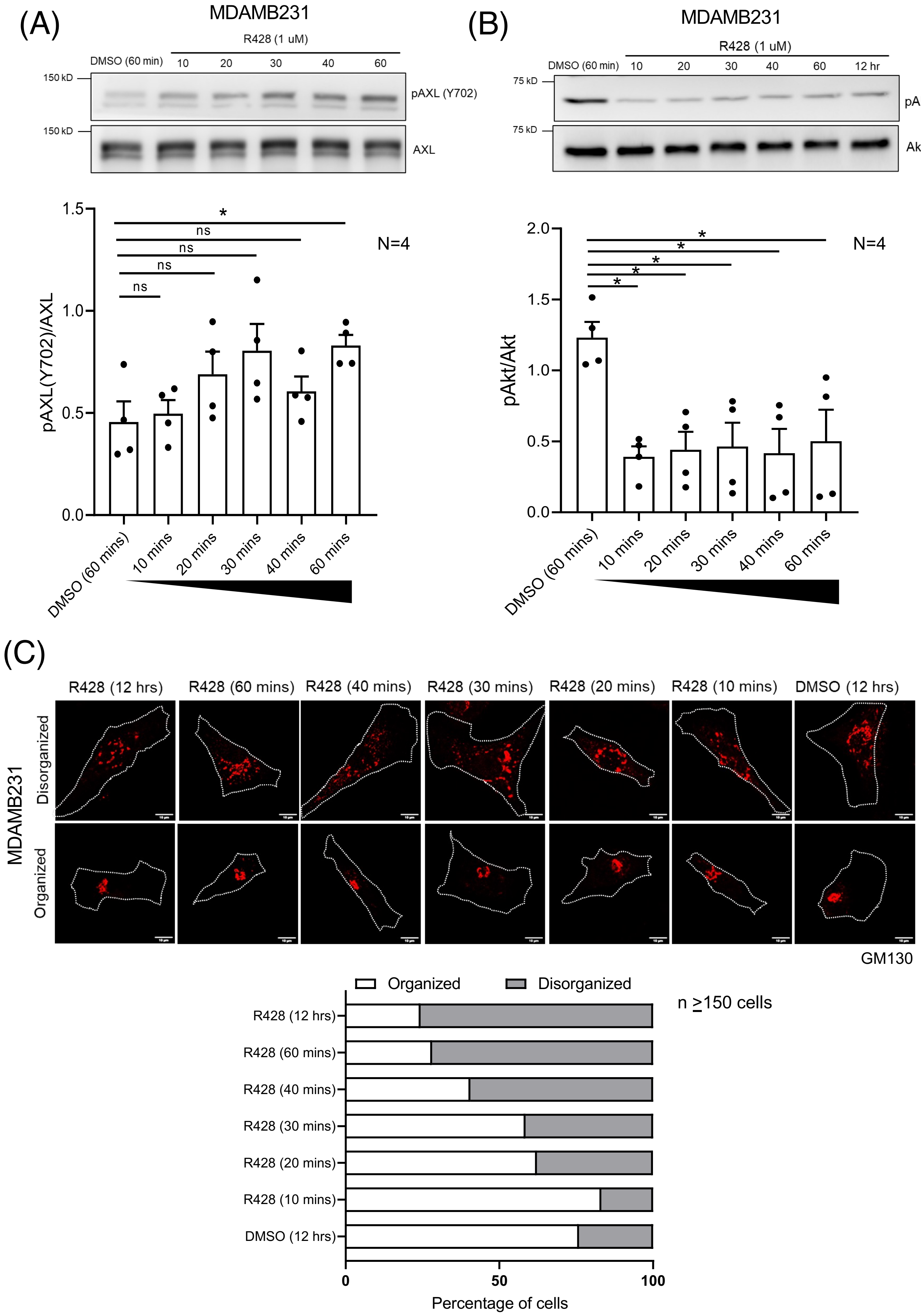

(A)
MDAMB231
R428 (1 uM)
DMSO (60 min)
10
20
30
40
60
150 kD
pAXL (Y702)
150 kD
AXL
pAXL(Y702)/AXL
N=4
ns
ns
ns
ns
*
1.5
1.0
0.5
0.0
DMSO (60 mins)
10 mins
20 mins
30 mins
40 mins
60 mins
(B)
MDAMB231
R428 (1 uM)
DMSO (60 min)
10
20
30
40
60
12 hr
75 kD
pAkt
75 kD
Akt
pAkt/Akt
N=4
*
*
*
*
*
2.0
1.5
1.0
0.5
0.0
DMSO (60 mins)
10 mins
20 mins
30 mins
40 mins
60 mins
(C)
R428 (12 hrs)
R428 (60 mins)
R428 (40 mins)
R428 (30 mins)
R428 (20 mins)
R428 (10 mins)
DMSO (12 hrs)
MDAMB231
Disorganized
Organized
GM130
Organized
Disorganized
n ≥150 cells
R428 (12 hrs)
R428 (60 mins)
R428 (40 mins)
R428 (30 mins)
R428 (20 mins)
R428 (10 mins)
DMSO (12 hrs)
0
50
100
Percentage of cells

(D)

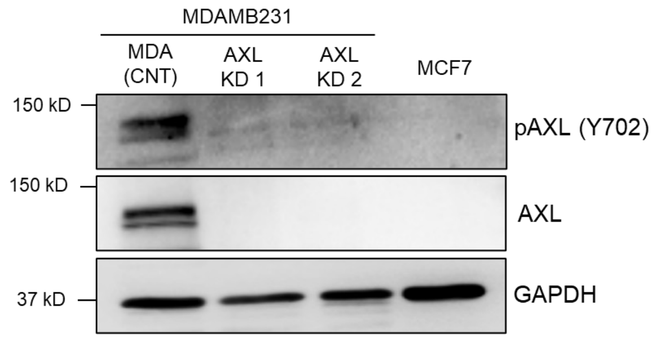

(E)

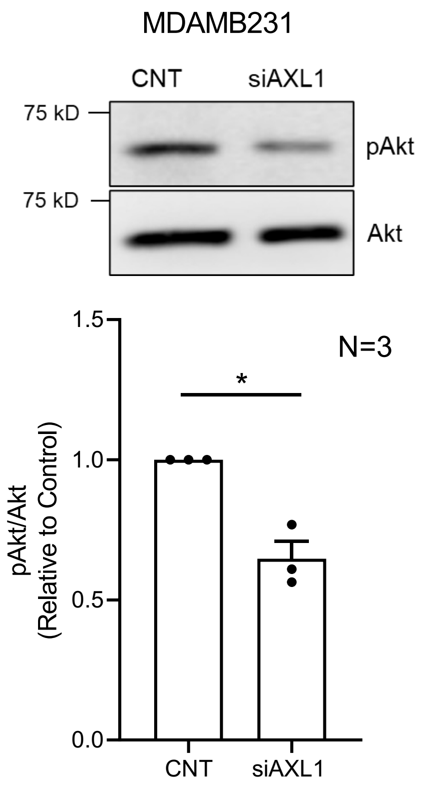

Fig. S3. AXL mediated regulation of Golgi organisation in adherent MDAMB231 cells. Representative western blots for **(A)** Y702 phosphorylated AXL (pAXL) and total AXL (AXL) and **(B)** phosphorylated Akt (pAkt) and total Akt (Akt) in cell lysates from DMSO treated for 60min (DMSO-60 min) and R428 treated for 10, 20, 30, 40, 60min MDAMB231 cells. The black bar below the graph represents the increasing time of R428 treatment. **(C)** Graph represents ratio of densitometric band intensities as mean±SEM from four independent experiments. presentative cross-section images of the organised and disorganised Golgi phenotype in GM130 (red) immunostained control (DMSO-12hrs) or R428 treated for 10, 20, 30, 40, 60 min and 12hrs MDAMB231 cells. Percentage distribution profile of cells (n≥150) showing organized (white) and disorganized (grey) Golgi across treatment conditions. Representative western blots for **(D)** Y702 phosphorylated AXL (pAXL), total AXL (AXL) and GAPDH in cell lysates from control MDAMB231 (MDA-CNT), siAXL1 (AXL KD1), siAXL2 (AXL KD2) and MCF7 cells. **(E)** Representative western blots for phosphorylated Akt (pAkt) and total Akt (Akt) in cell lysates from control (CNT) and siAXL1 treated MDAMB231 cells. Graph represents ratio of densitometric band intensities (normalized to CNT) as mean±SEM from three independent experiments. Statistical analysis was done using Mann-Whitney U test for non-normalised and single sample t test for normalised data. Scale bars are 10μm. (*p≤0.05, **p≤0.01, ***p≤0.001, ****p≤0.0001, ns=not significant).

Biology Open • Supplementary information

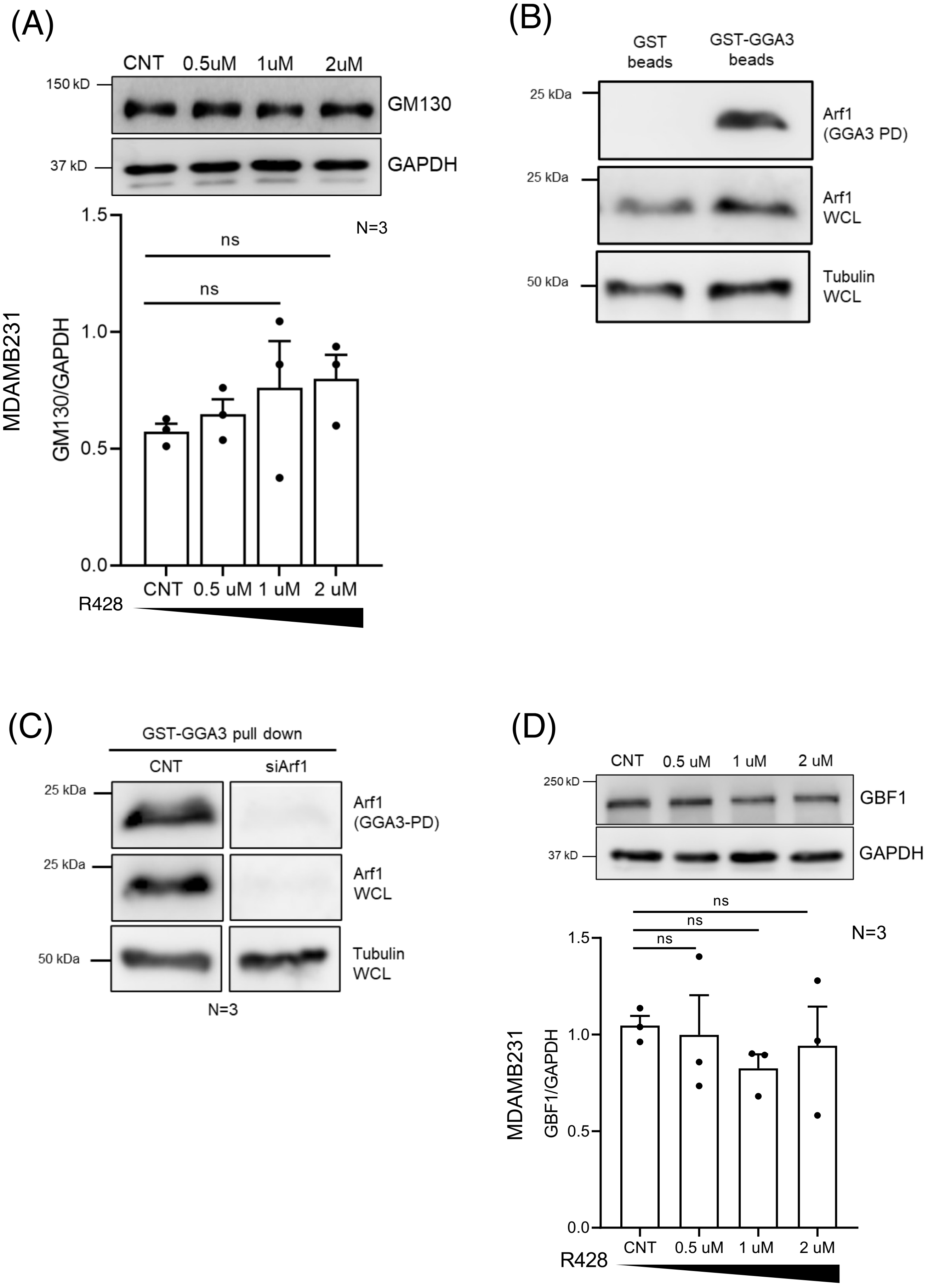

Biology Open • Supplementary information

(E)

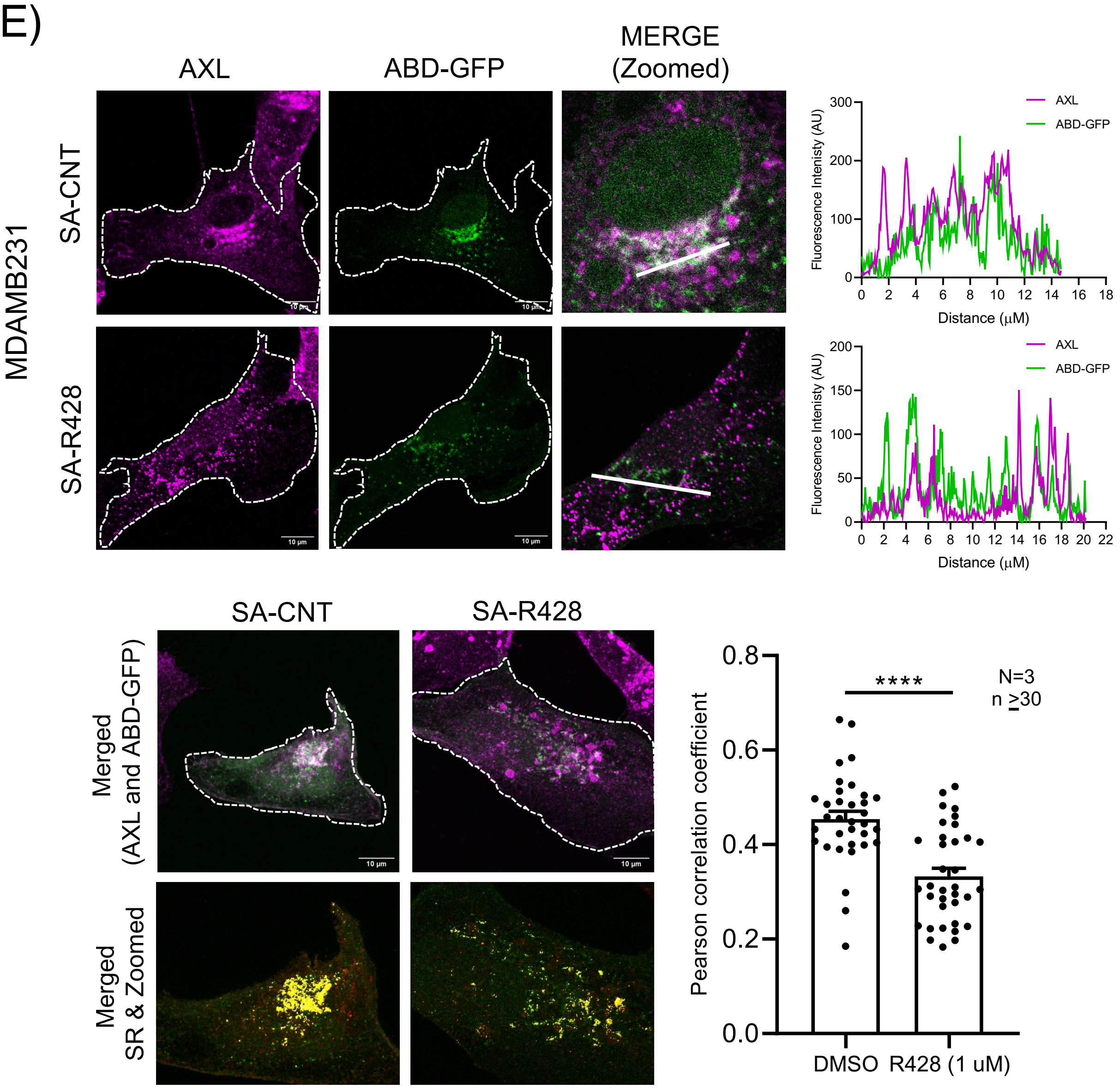
AXL
ABD-GFP
MERGE (Zoomed)
MDAMB231
SA-CNT
SA-R428
Fluorescence Intenisty (AU)
Distance (µM)
AXL
ABD-GFP
SA-CNT
SA-R428
Merged (AXL and ABD-GFP)
Merged SR & Zoomed
Pearson correlation coefficient
****
N=3
n ≥30
DMSO
R428 (1 uM)

(F)

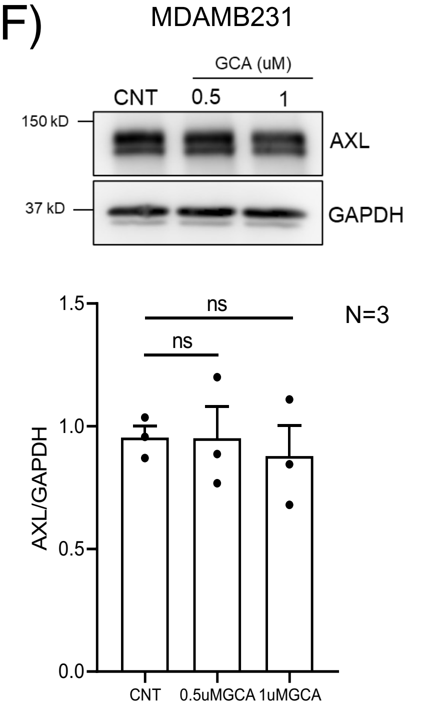
MDAMB231
GCA (uM)
CNT
0.5
1
150 kD
AXL
37 kD
GAPDH
AXL/GAPDH
ns
ns
N=3
CNT
0.5uMGCA
1uMGCA

(G)

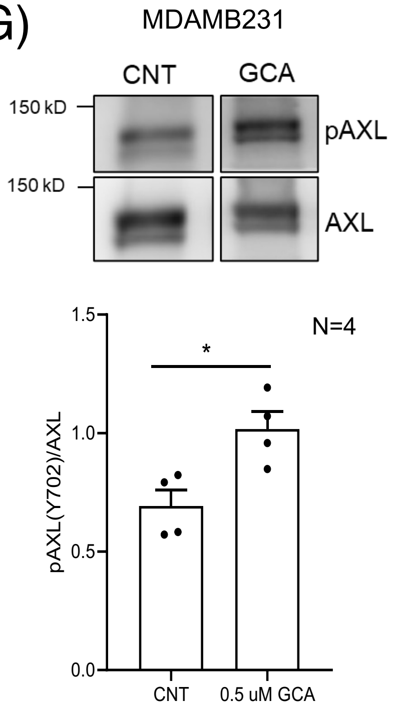
MDAMB231
CNT
GCA
150 kD
pAXL
150 kD
AXL
pAXL(Y702)/AXL
*
N=4
CNT
0.5 uM GCA

Fig. S4. AXL-Arf1 axis regulates Golgi organisation in adherent MDAMB231 cells (A) Representative western blot for GM130 and GAPDH levels in adherent MDAMB231 cells treated with DMSO (CNT) or R428 (0.5μM, 1μM and 2μM). The graph represents ratio of densitometric band intensities as mean±SEM from three independent experiments. **(B)** Representative immunoblots showing detection of Arf1 in GST-only bead controls, GST-GGA3 pulldown fractions (active Arf1-bound), and corresponding whole-cell lysates (WCL). **(C)** Representative immunoblots for Arf1 detection in GST-GGA3 pulldown fractions, and corresponding whole-cell lysates (WCL) in control and Arf1 knockdown MDAMB231 cell lysates (N=3). **(D)** Representative western blot for GBF1 and GAPDH levels in adherent MDAMB231 cells treated with DMSO (CNT) or R428 (0.5μM, 1μM, 2μM). The graph represents ratio of densitometric band intensities as mean±SEM from three independent experiments. The black bar below any graph represents the gradient of increasing R428 concentration **(E)** Representative deconvoluted images with merged zoom insets and surface rendering (SR) with line plots for stable adherent (SA) MDAMB231 cells expressing ABD-GFP (green) and immunostained for AXL (magenta), treated with DMSO (SA-CNT) or R428 (SA-R428). Graph represents Pearson's coefficients for ABD-GFP (green) and AXL (magenta) colocalization plotted as mean ± SEM for n ≥30 cells for three experiments. The SR zoom image represents the colocalization overlap marked in yellow. **(F)** Representative western blots for AXL and GAPDH in DMSO (CNT), 0.5μM and 1μM GCA treated MDAMB231 cell lysates. Graph represents ratio of densitometric band intensities as mean±SEM from three independent experiments. **(G)** Representative western blots for Y702 phosphorylated AXL (pAXL) and total AXL in DMSO (CNT), 0.5μM GCA treated MDAMB231 cell lysates. Graph represents ratio of densitometric band intensities as mean±SEM from four independent experiments. Statistical analysis was done using Mann-Whitney U test for non-normalised western blotting results. Statistical analysis was done using one-way ANOVA multiple comparisons test with Tukey's method for error correction for the distribution profile and for Pearson's colocalization analysis. Scale bars are 10 μm. (*p≤0.05, **p≤0.01, ***p≤0.001, ****p≤0.0001, ns=not significant).

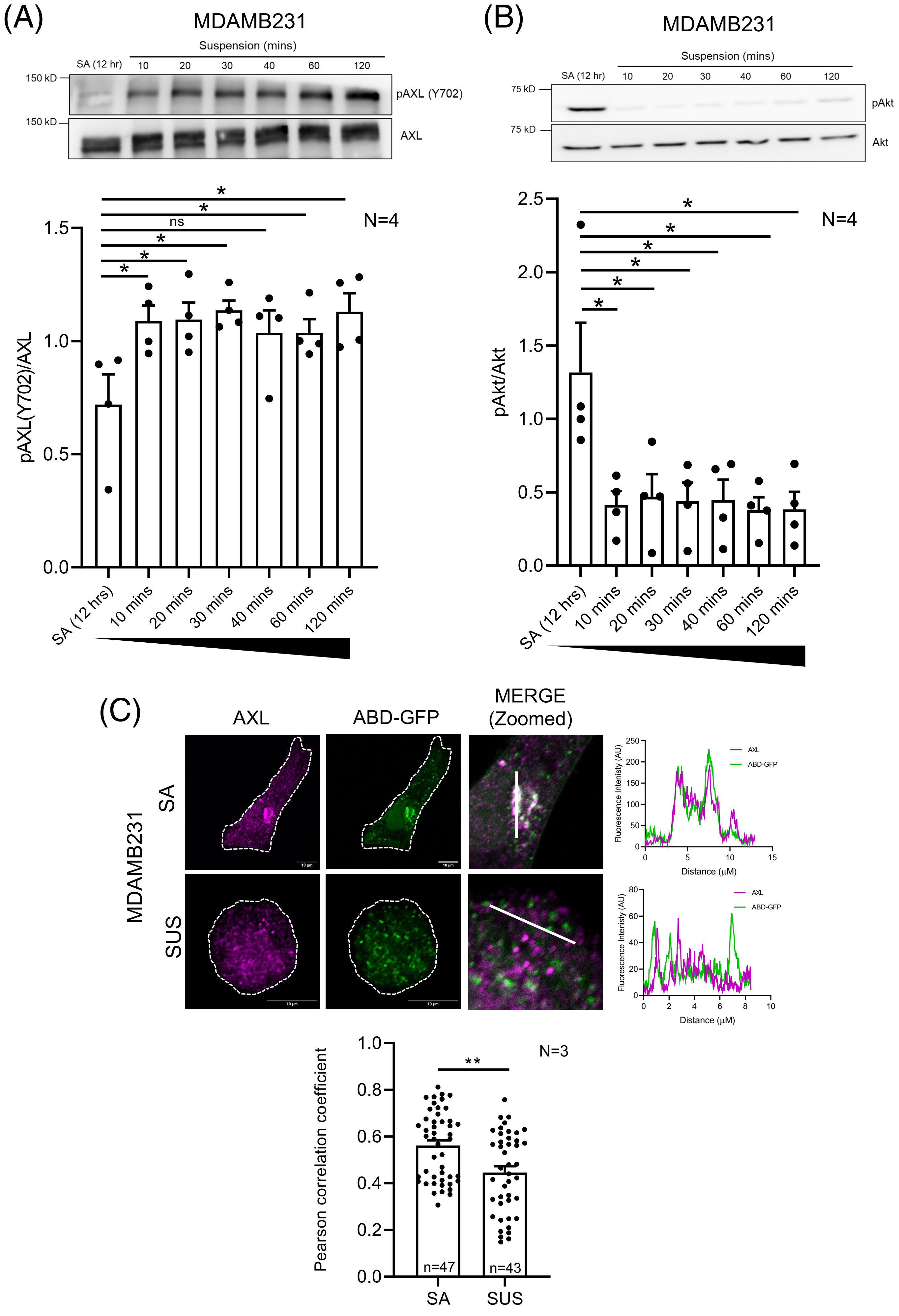

Biology Open • Supplementary information

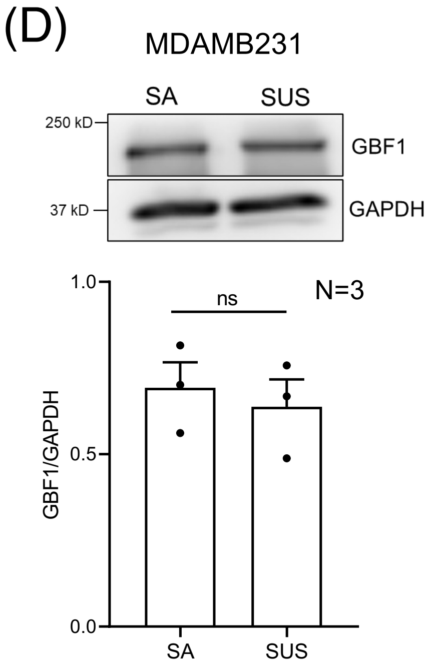

Fig. S5. Role of AXL-Arf1 axis in loss of adhesion mediated Golgi disorganisation in MDAMB231 cells. **(A-B)** Representative western blots for **(A)** Y702 phosphorylated AXL (pAXL) and total AXL (AXL) and **(B)** phosphorylated Akt (pAkt) and total Akt (Akt) in cell lysates from stable-adherent (SA) and non-adherent MDAMB231 cells suspended for 10, 20, 30, 40, 60 and 120 minutes. The black bar below the graph represents the increasing time of R428 treatment. The graphs represent the ratio of densitometric band intensities as mean±SEM from four independent experiments. **(C)** Representative cross-section images with merged zoom insets and line plots for stable adherent (SA) and non-adherent (SUS) MDAMB231 cells expressing ABD-GFP (green) and immunostained for AXL (magenta). Graph represents Pearson's coefficients for AXL (magenta) and ABD-GFP (green) colocalization plotted as mean ± SEM for n ≥40 cells from three experiments **(D)** Representative western blot for GBF1 and GAPDH in lysates from stable-adherent (SA) and non-adherent (SUS) MDAMB231 cells. The graphs represent ratio of densitometric band intensities as mean±SEM from three independent experiments. Statistical analysis was done using Mann-Whitney U test for non-normalised western blotting results. Scale bars are 10μm. (*p≤0.05, **p≤0.01, ***p≤0.001, ****p≤0.0001, ns=not significant).

Biology Open • Supplementary information

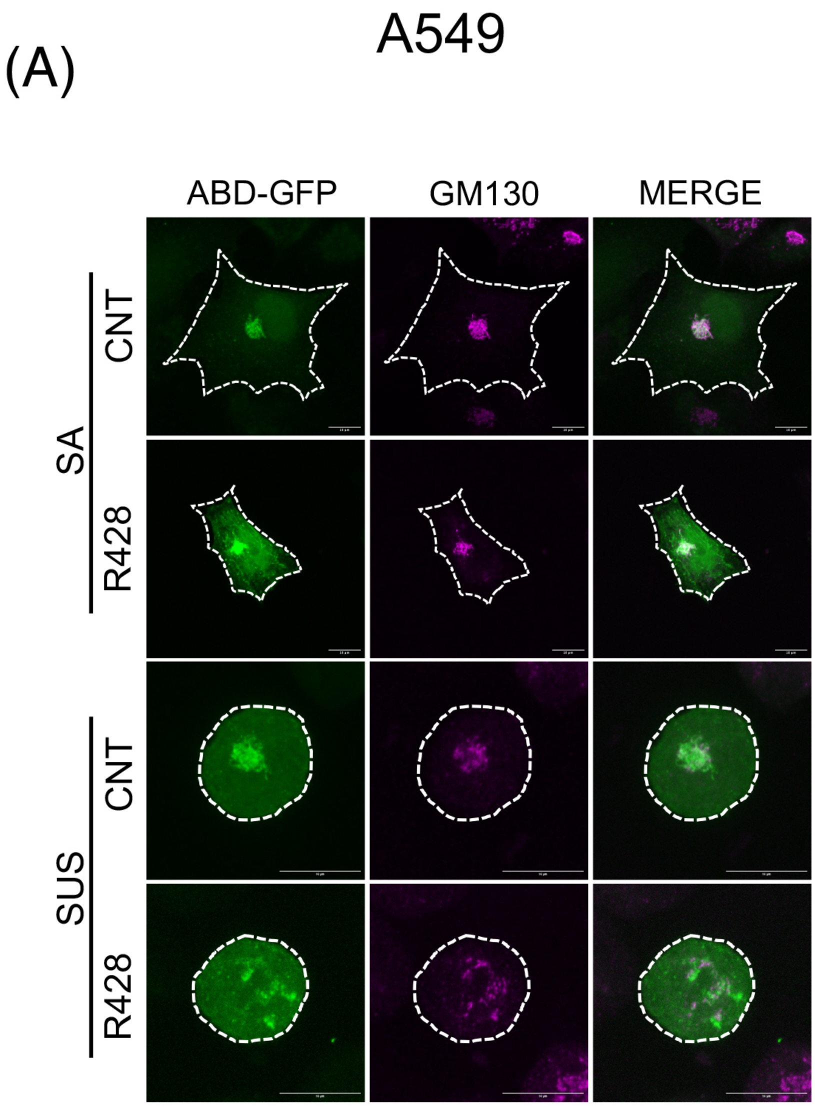

Fig. S6. R428 treatment and its effect on active Arf1 localization at the Golgi (A) A549 cells expressing ABD-GFP (green) to detect active Arf1 enriched at an intracellular location, seen to overlap with the Golgi, immunostained for GM130 (magenta) in adherent (SA) and non-adherent (SUS) cells treated with DMSO (CNT) or R428. Representative cross-section confocal images showing the predominant phenotype. Scale bars are 10μm.

Biology Open • Supplementary information

Blot transparency

Figure 2

(C)

AXL

Tubulin

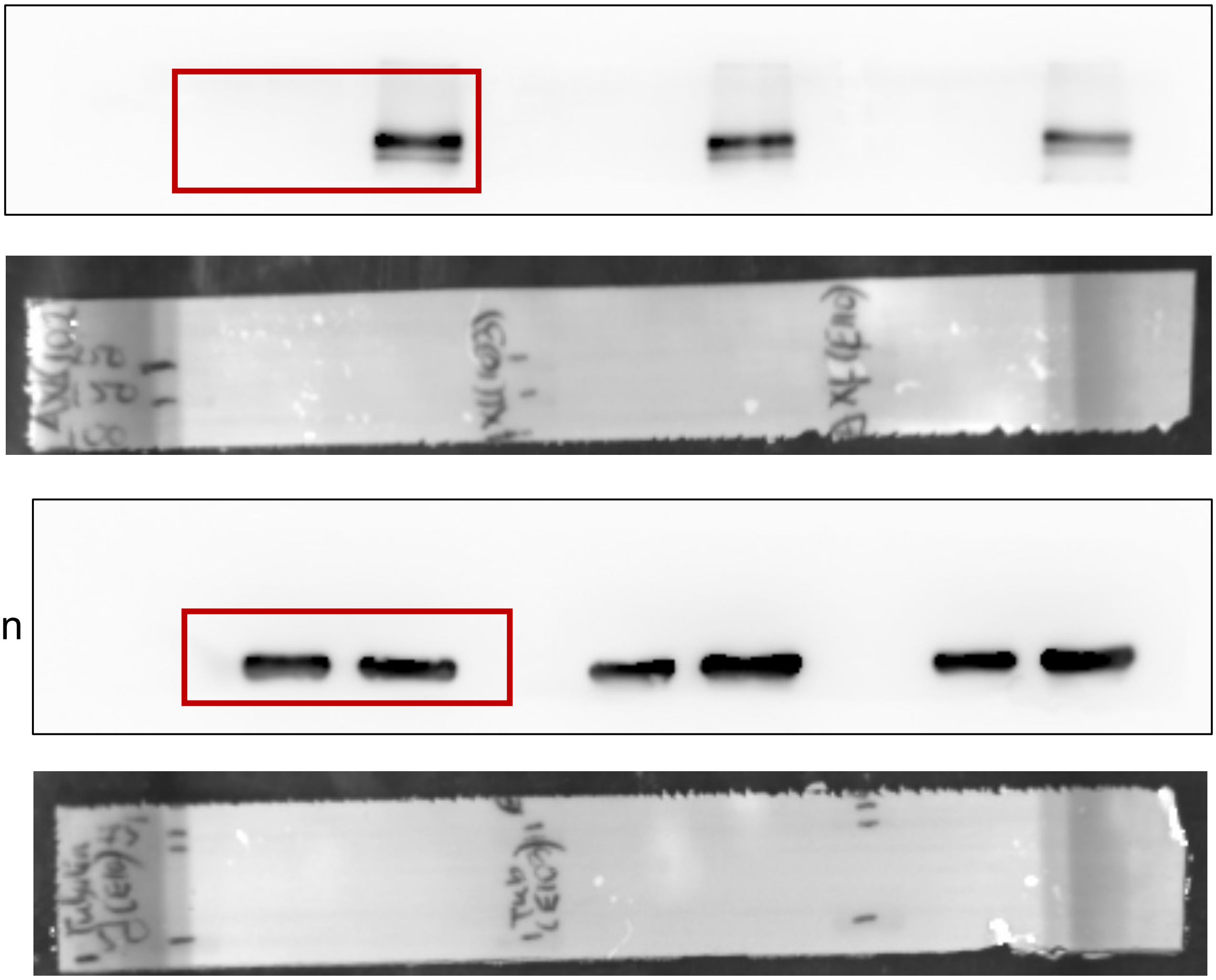

(D)

AXL

β-Tubulin

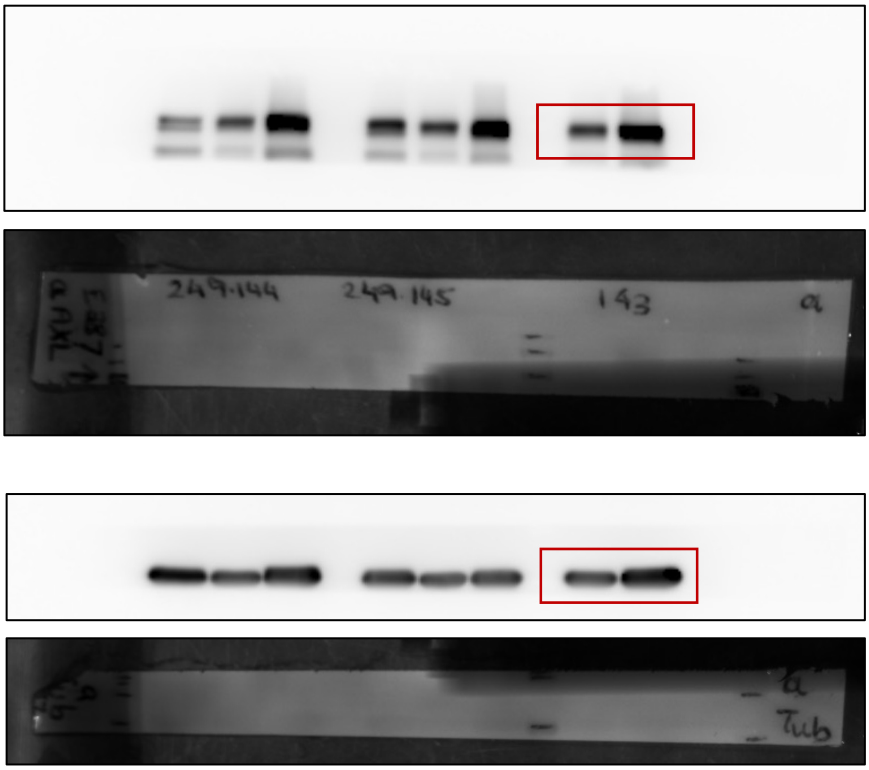

Biology Open • Supplementary information

Figure 3

(C)

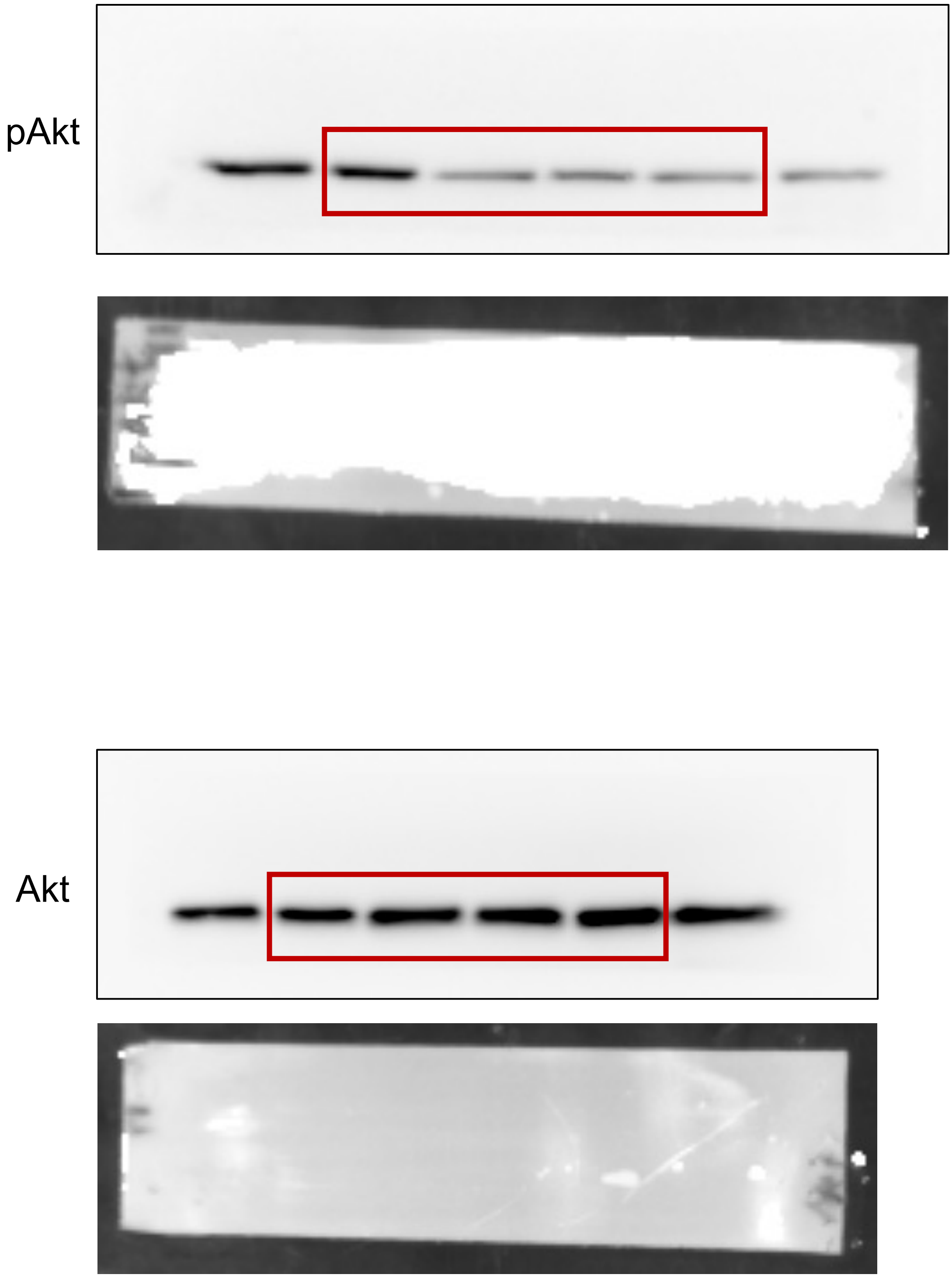

Biology Open • Supplementary information

Figure 3

(D)

pAXL

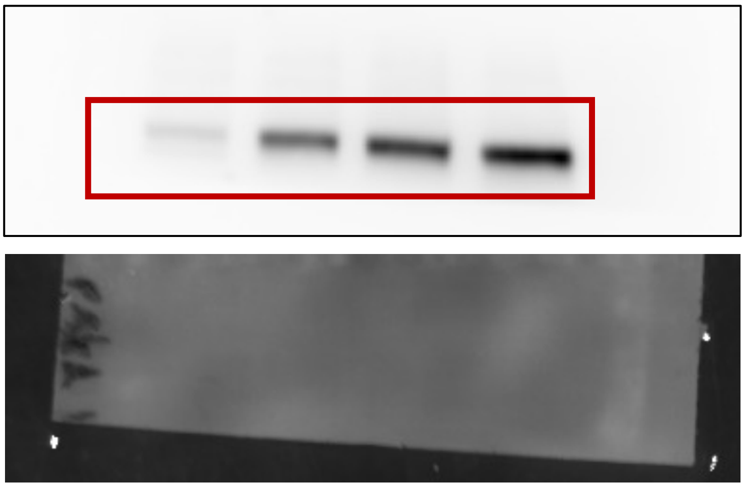

AXL

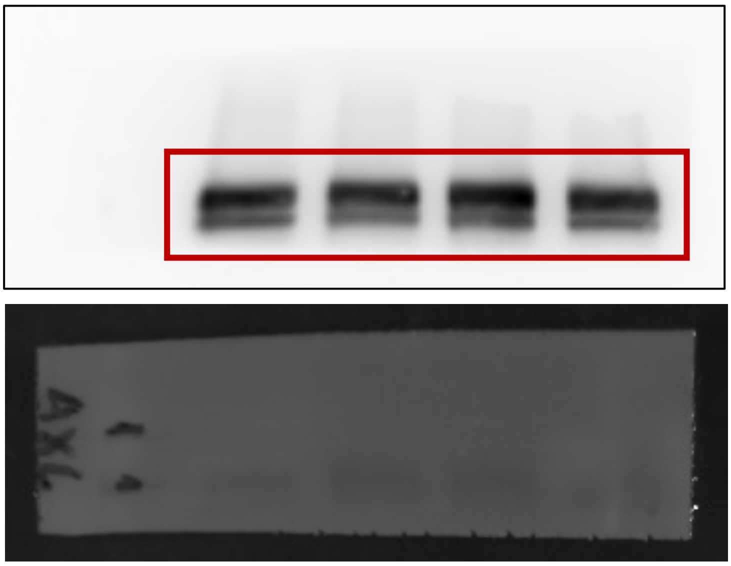

(F)

pAkt

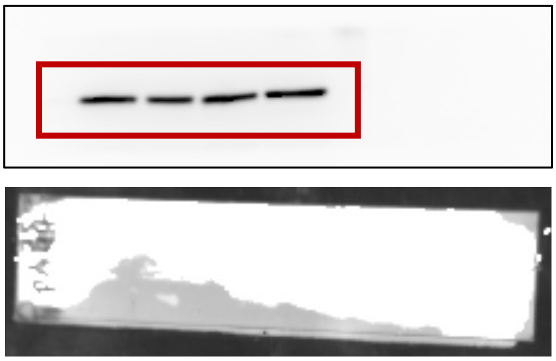

Akt

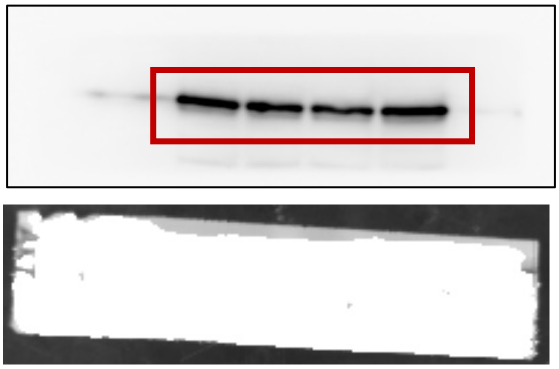

Biology Open • Supplementary information

Figure 3

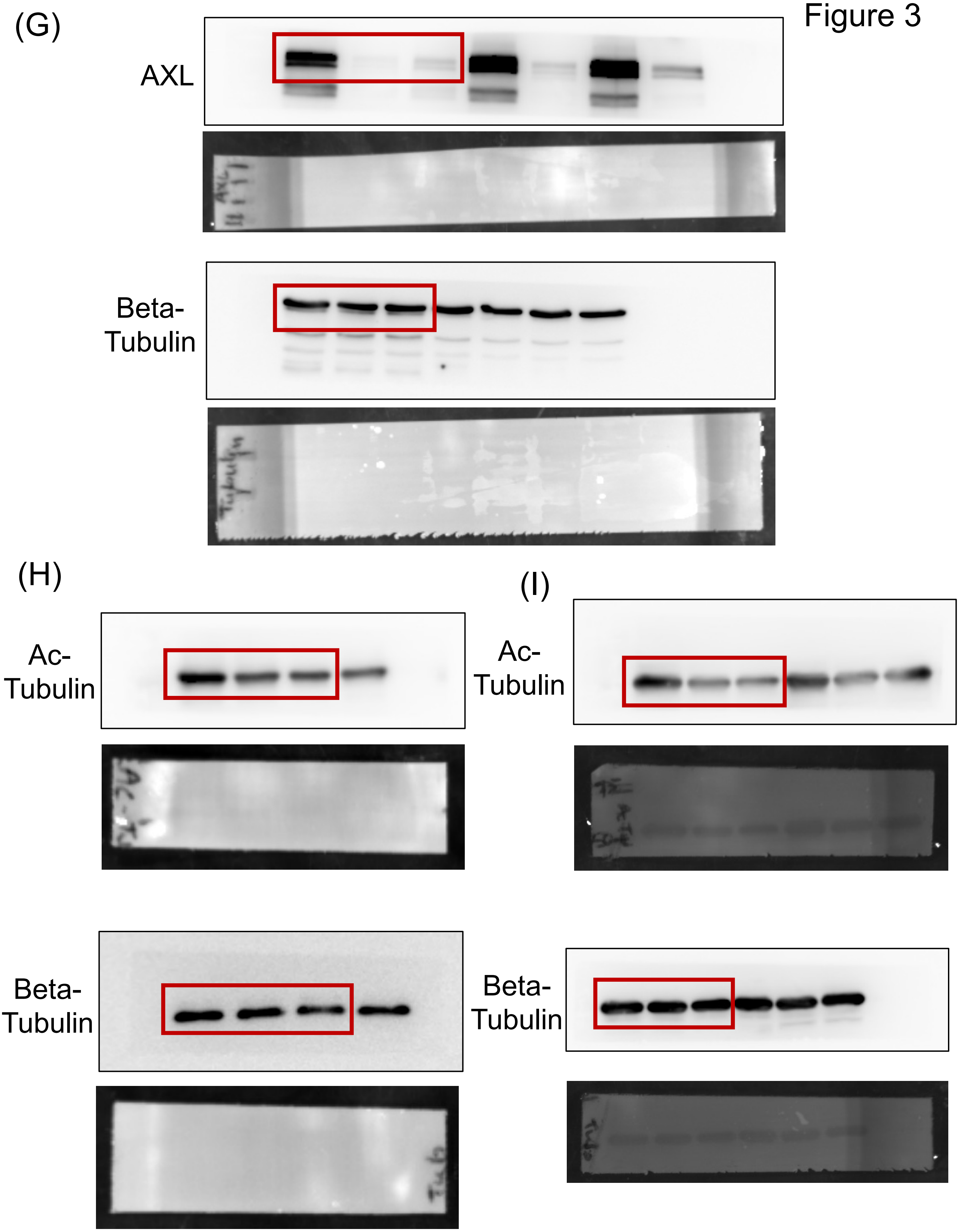

Biology Open • Supplementary information

(B)

Figure 4

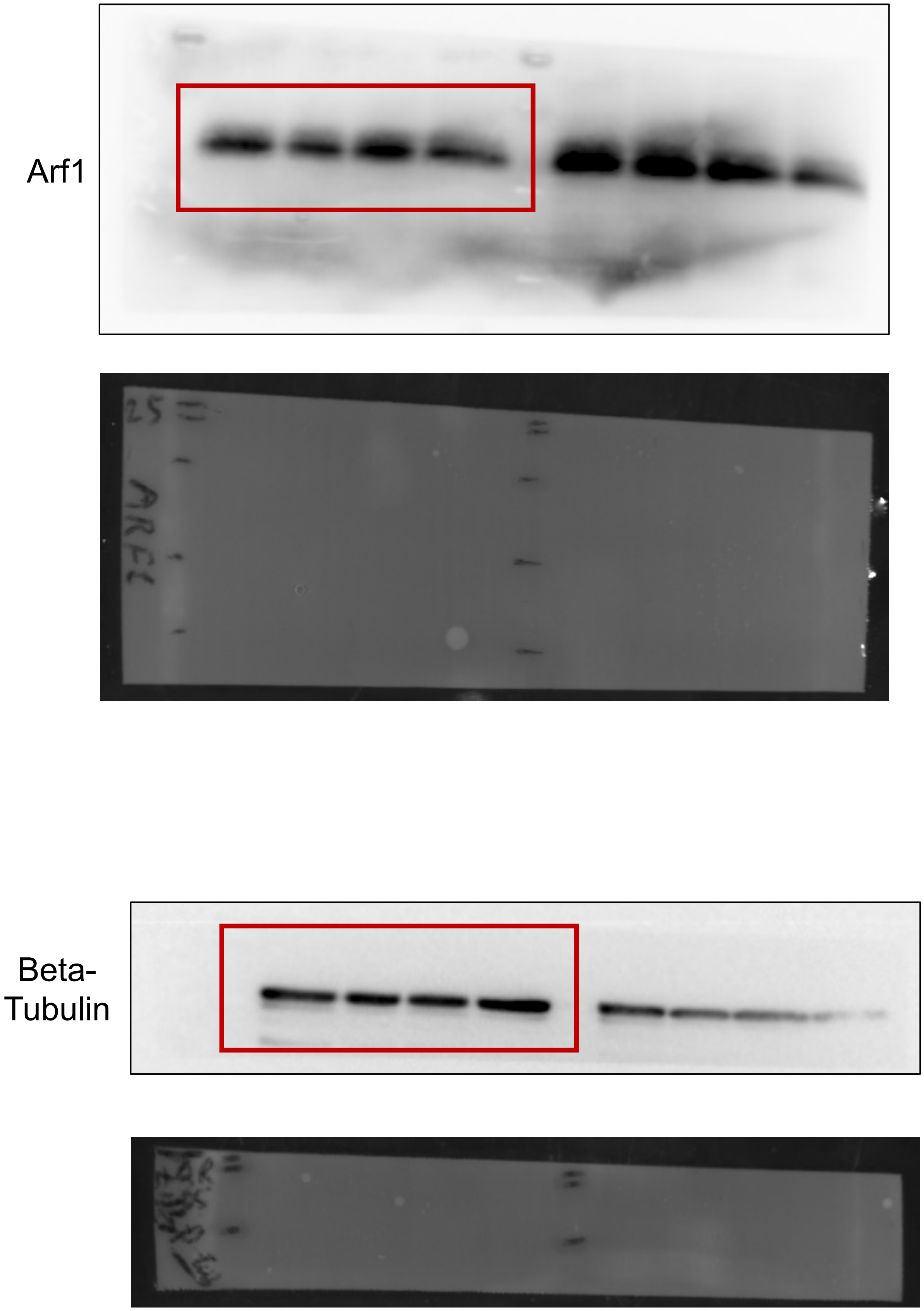

Biology Open • Supplementary information

Figure 4

(C)

(D)

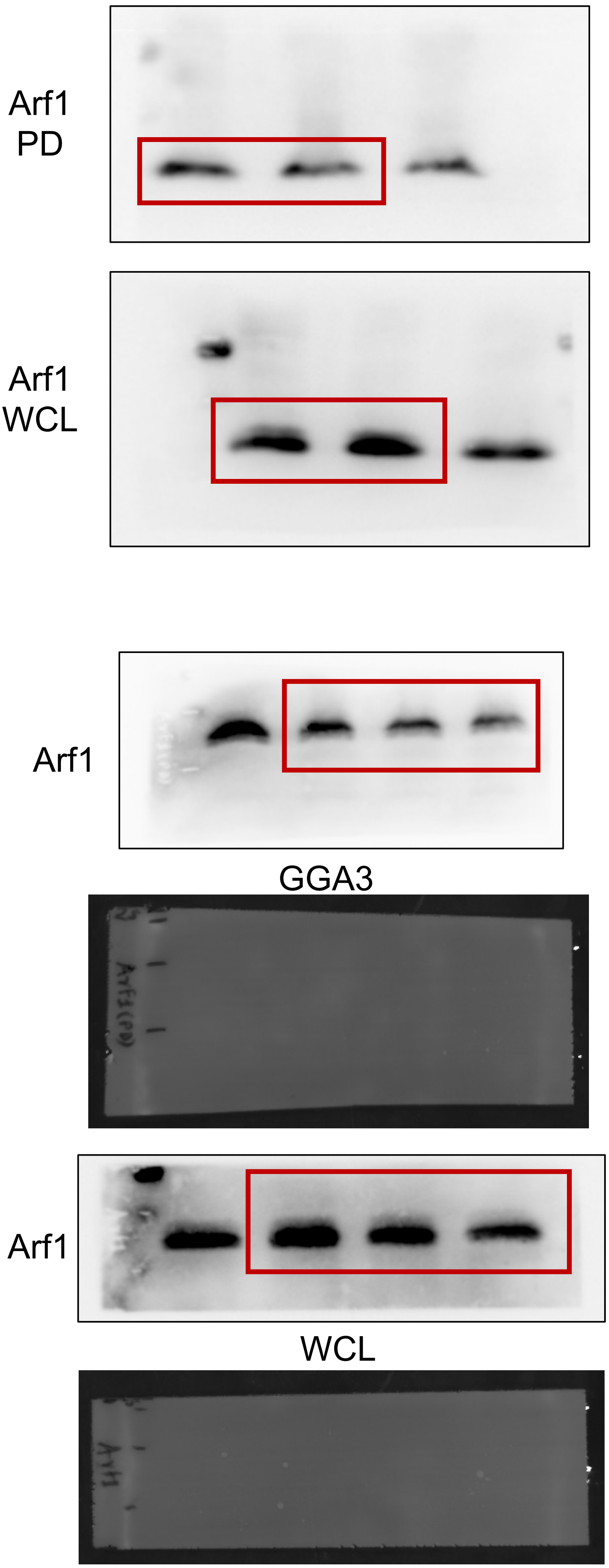

Figure 4

(I)

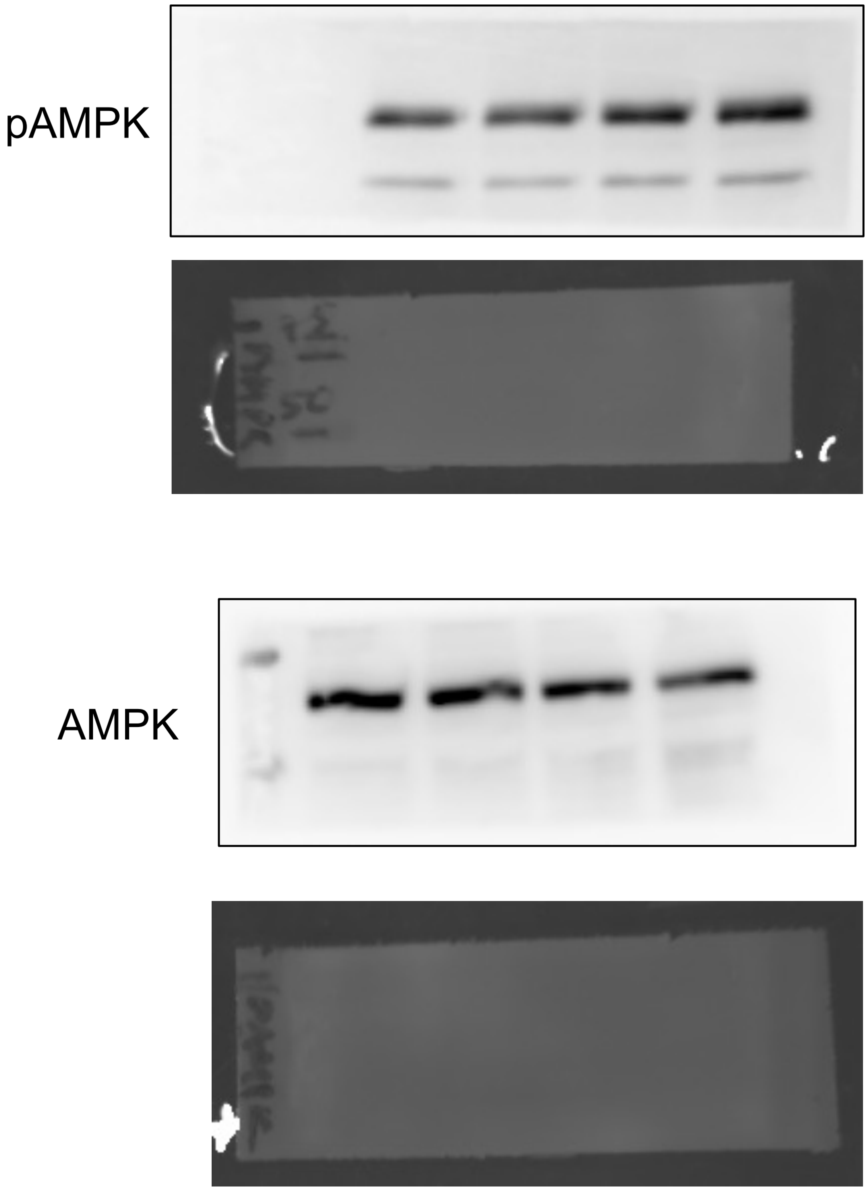

Biology Open • Supplementary information

Figure 5

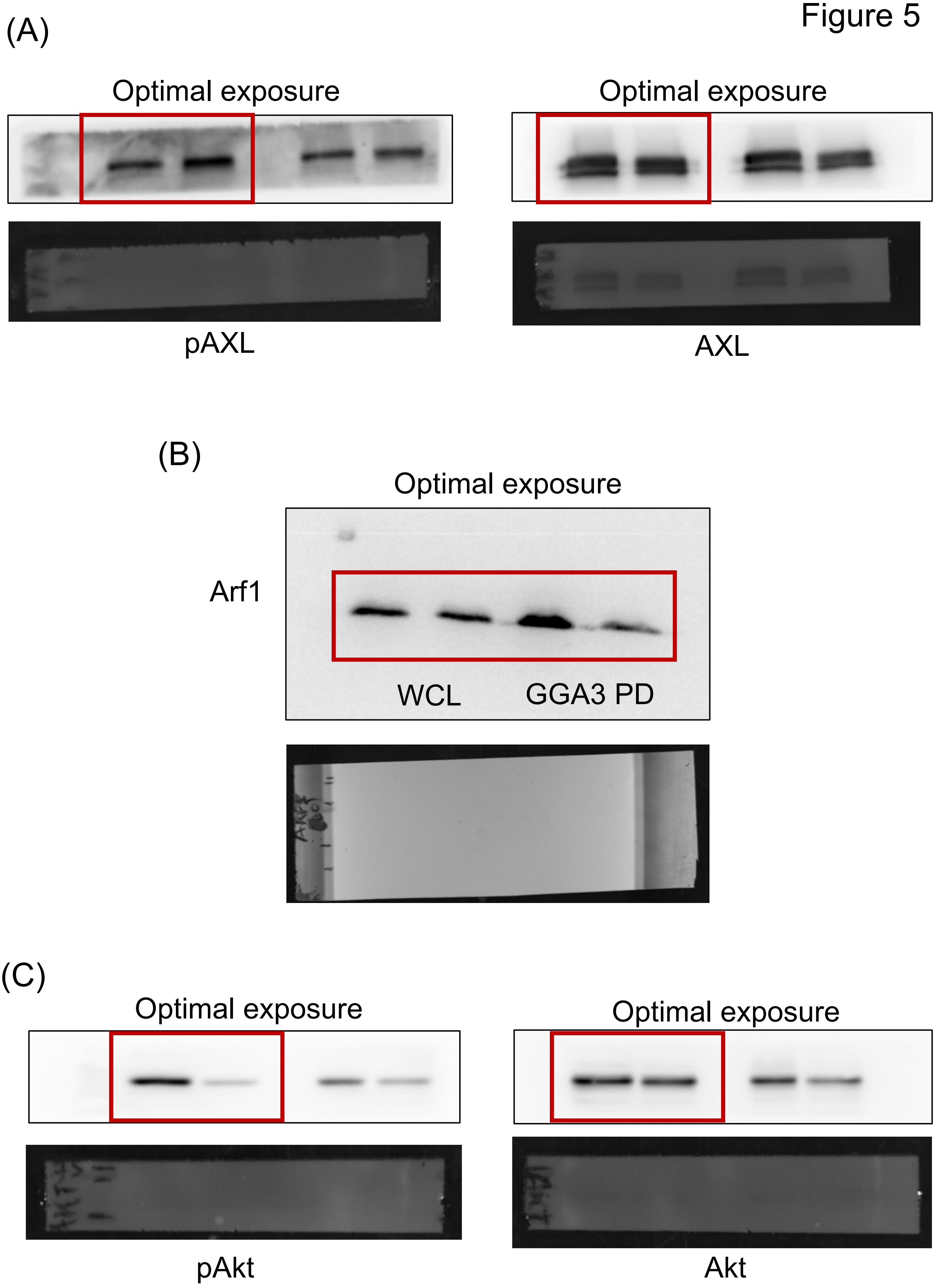

Biology Open • Supplementary information

Figure 5

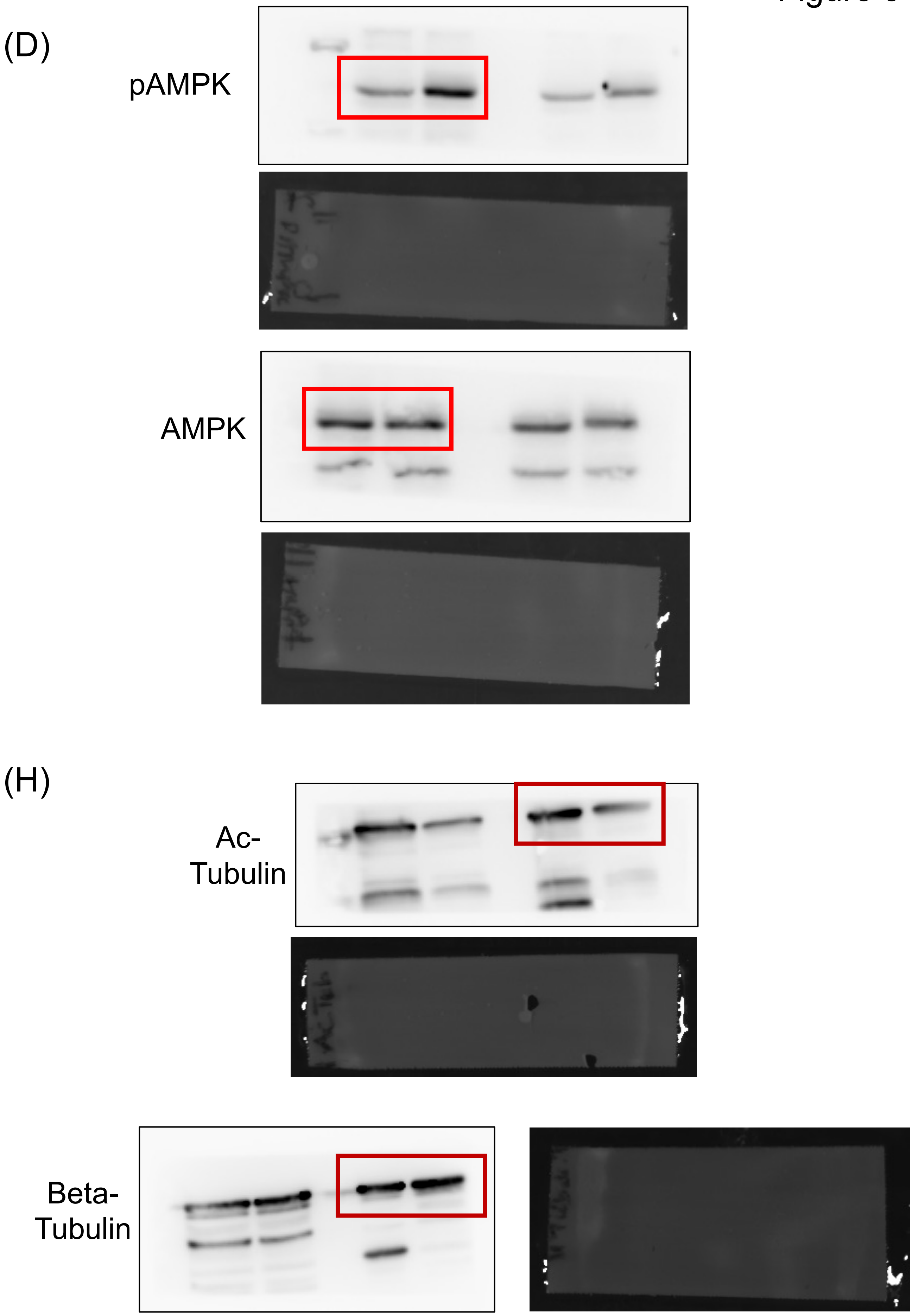

Figure 6

Figure 6C

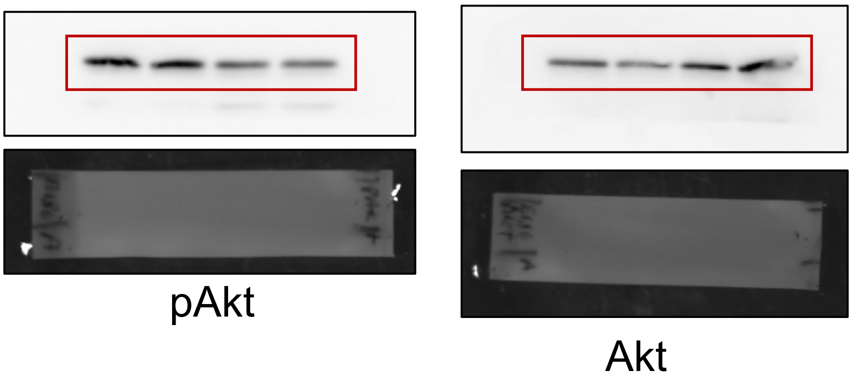

Figure 6D

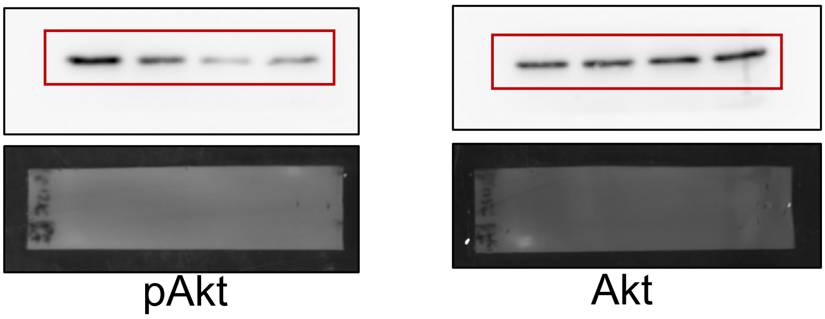

Figure 6E

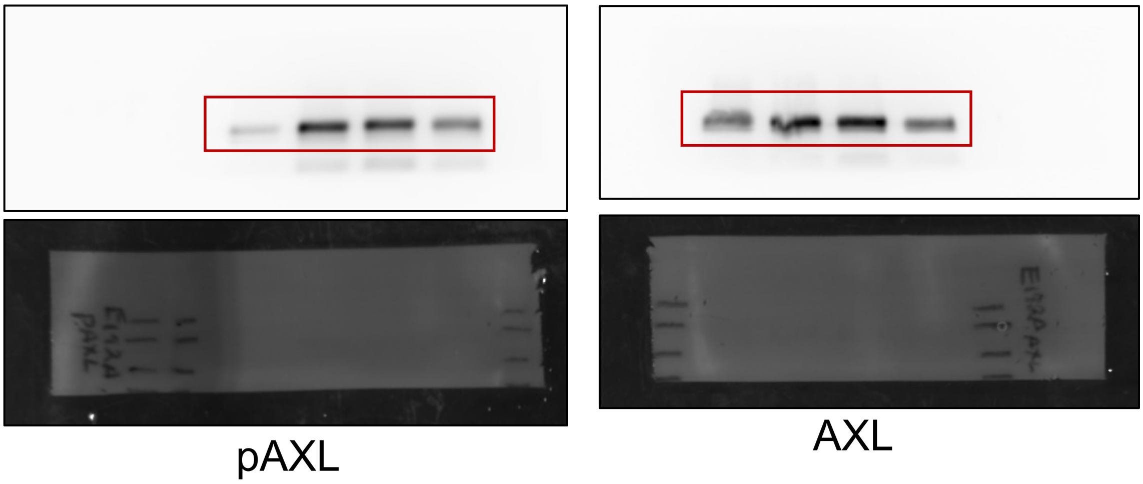

Figure 6F

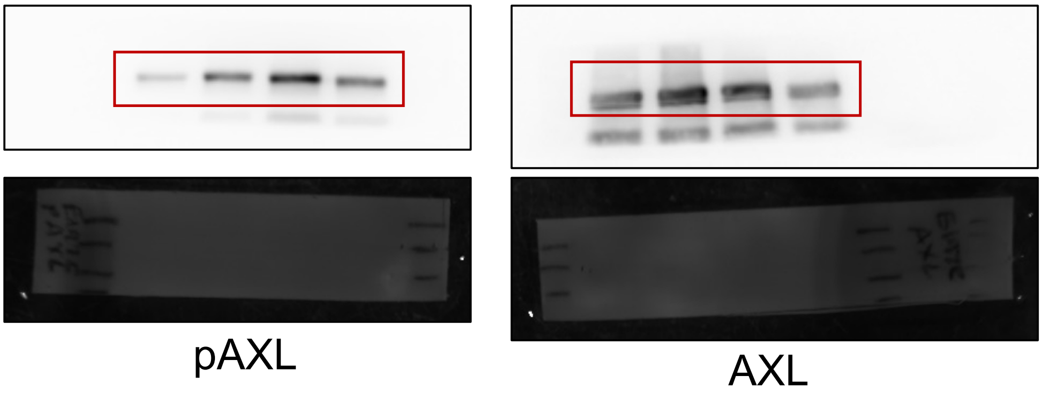

Figure 6

Figure 6H

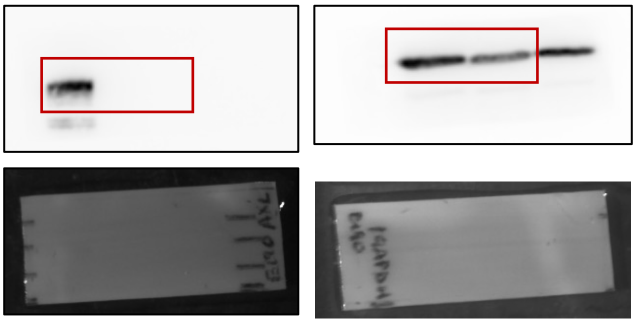

AXL GAPDH

Figure 6I

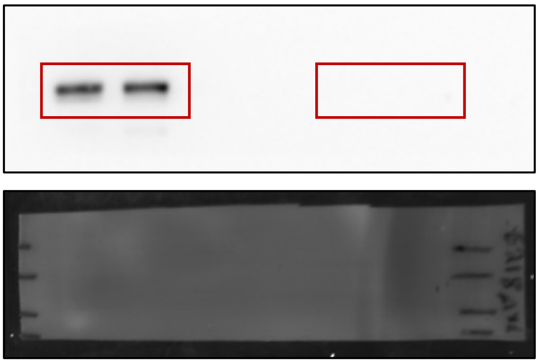

AXL

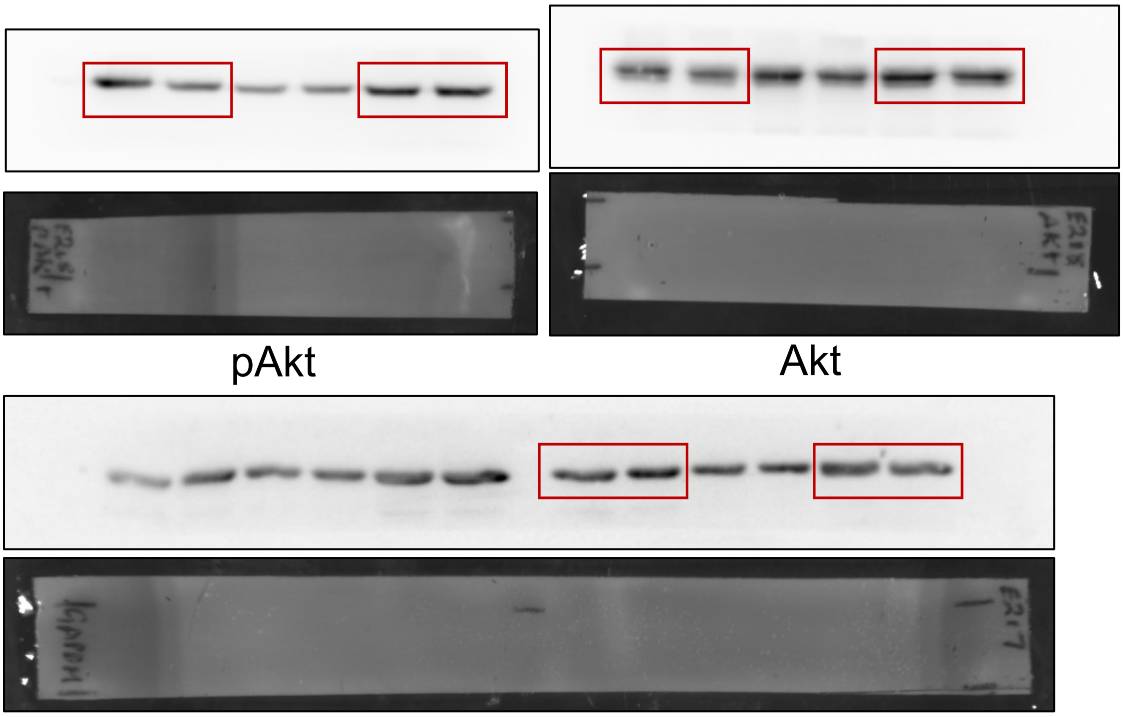

pAkt Akt

GAPDH

Biology Open • Supplementary information

Figure 7

Figure 7A

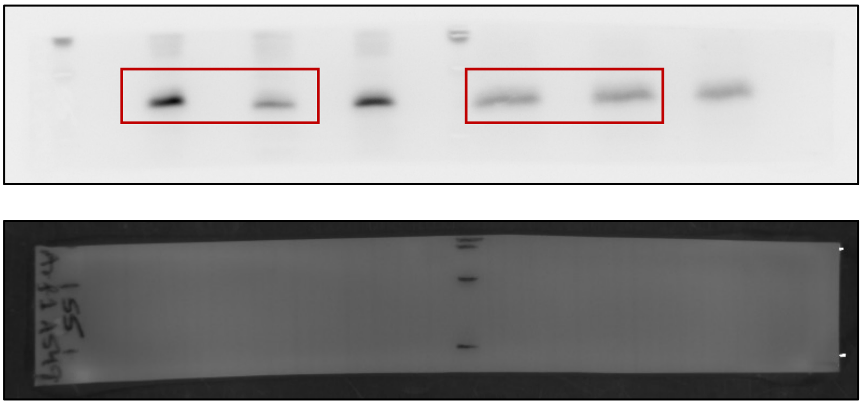

Arf1

Figure 7B

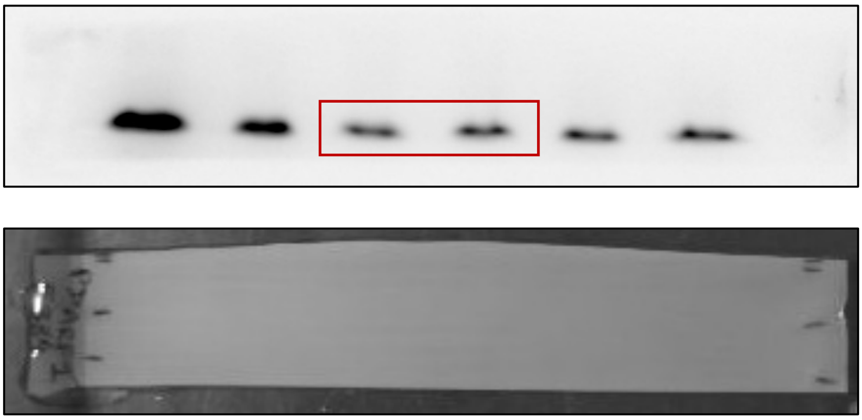

Arf1

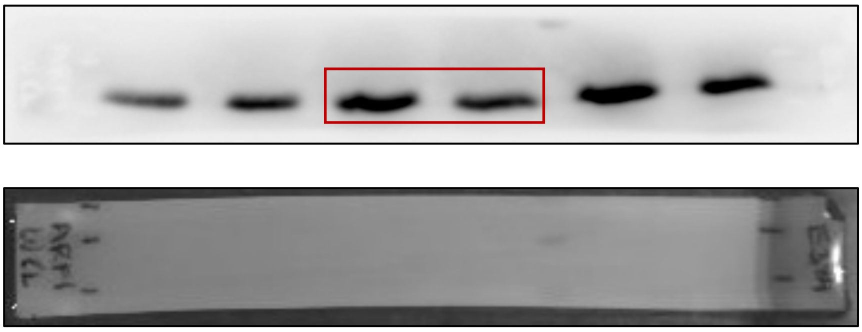

Arf1

Biology Open • Supplementary information

Biology Open (2026): doi:10.1242/bio.062581: Supplementary information

Figure 7

Figure 7C

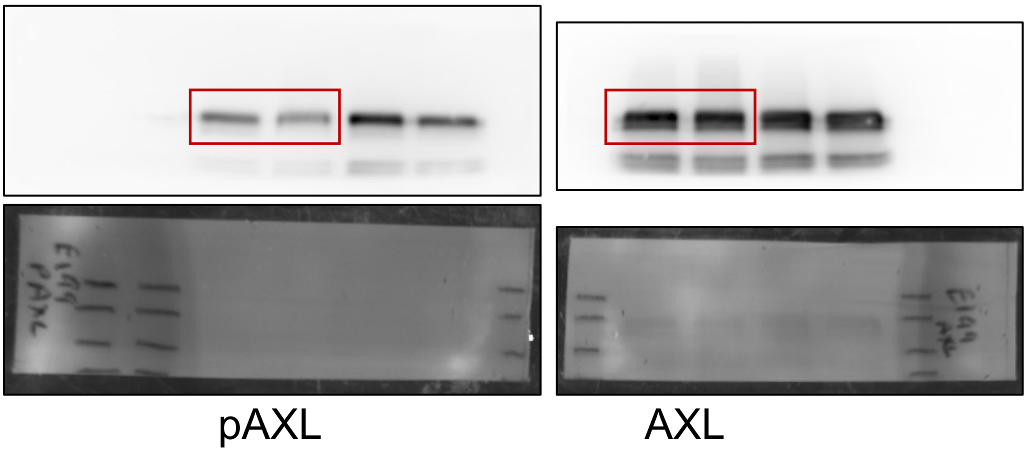

Figure 7D

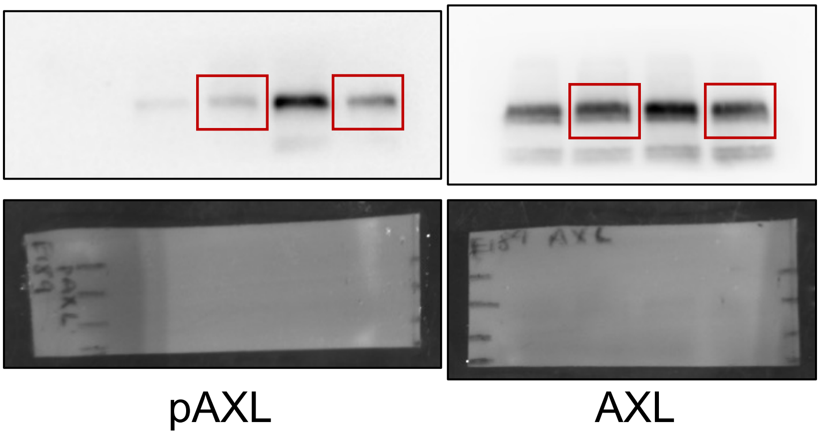

Figure 7E

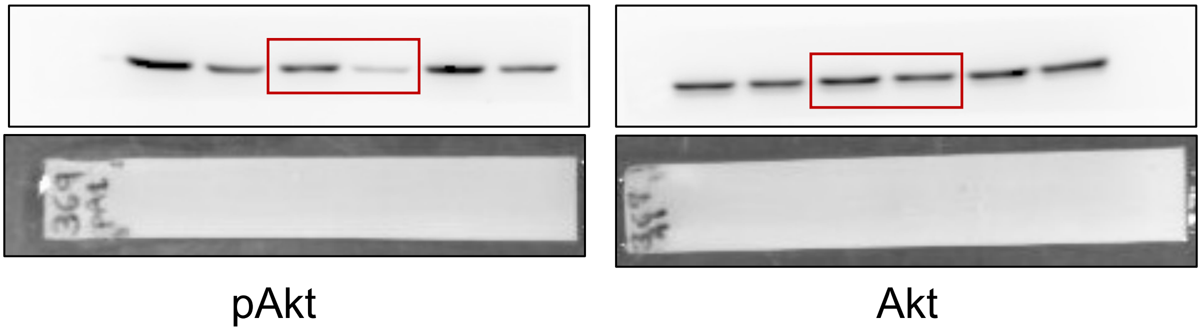

Biology Open • Supplementary information

Figure 7

Figure 7I

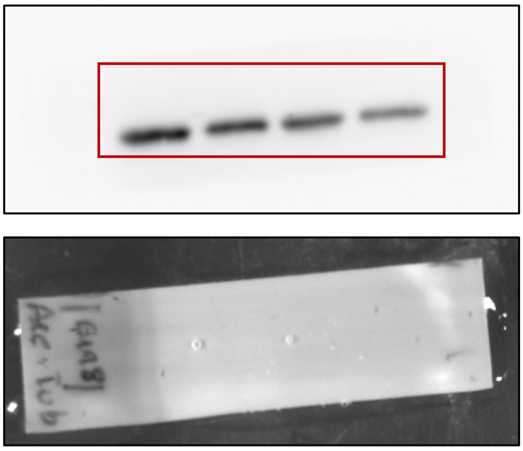

Acetylayed Tubulin

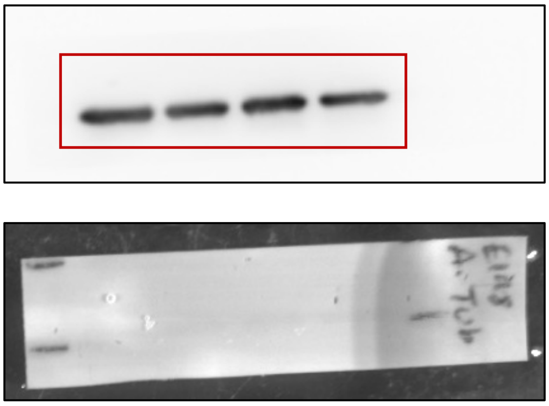

Total Tubulin

Biology Open • Supplementary information

Figure S3

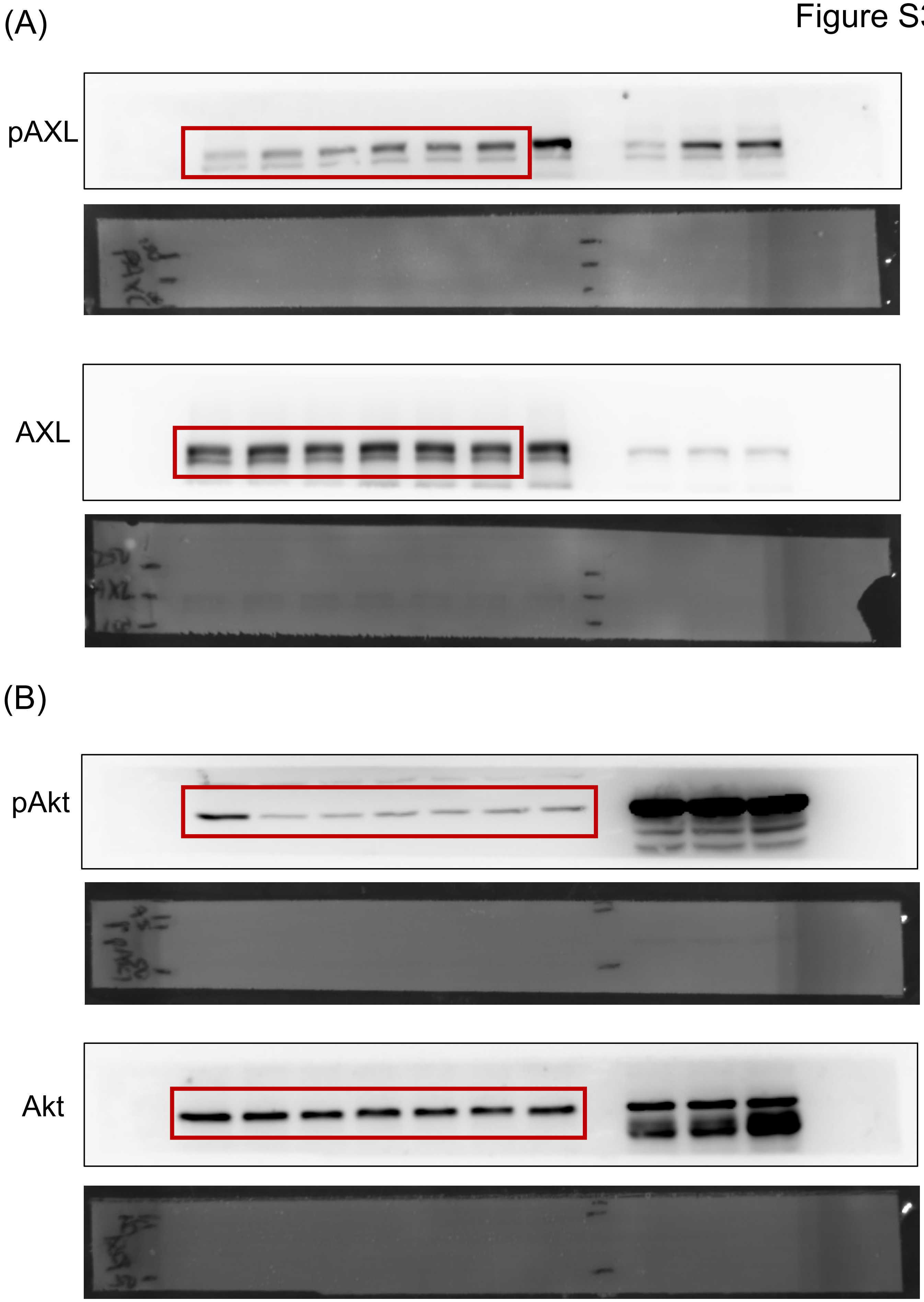

Biology Open • Supplementary information

Figure S3

(D)

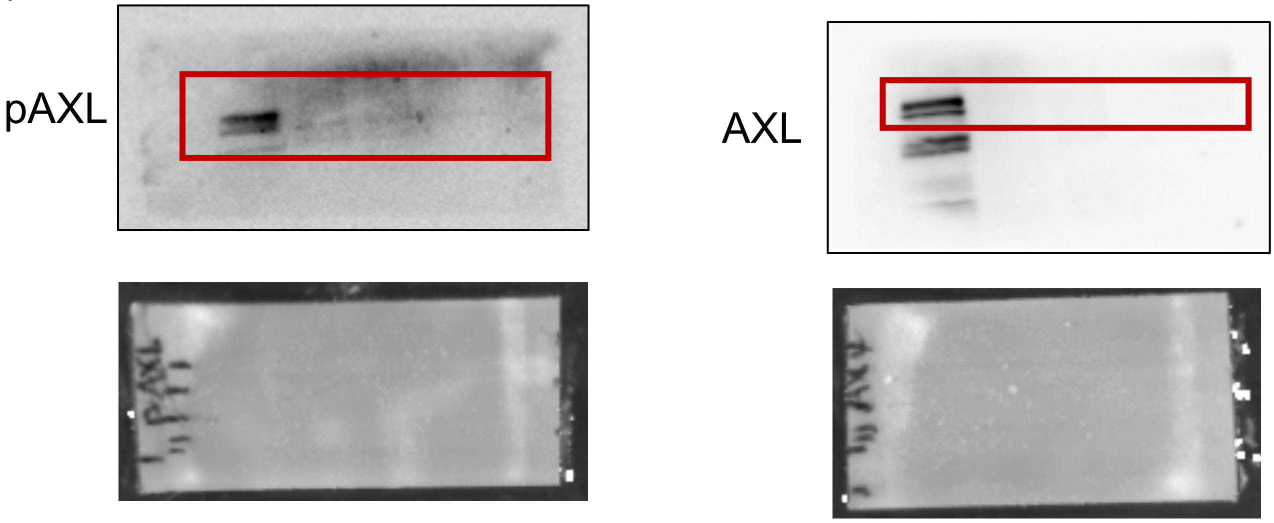

(E)

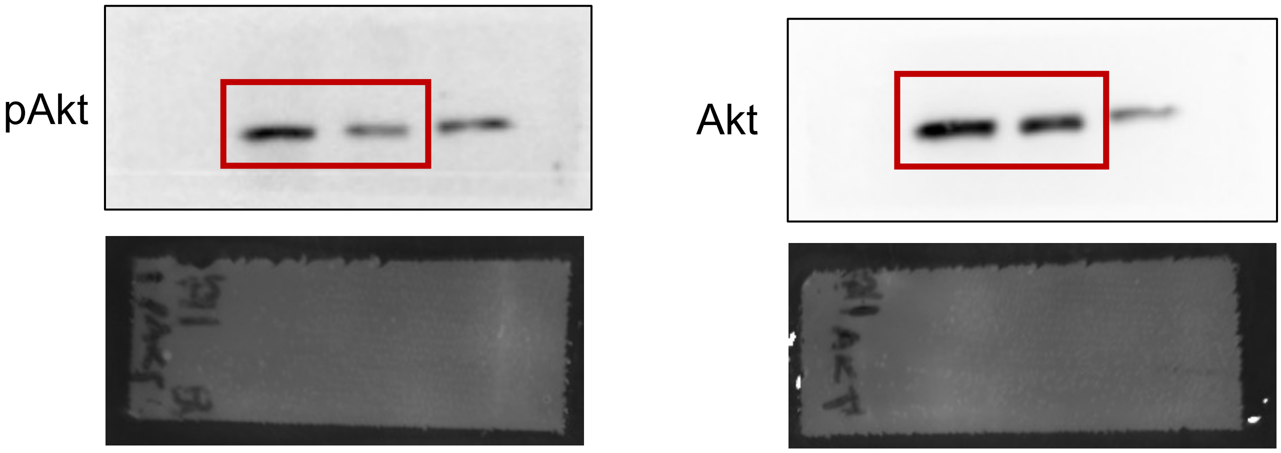

Figure S4

(A)

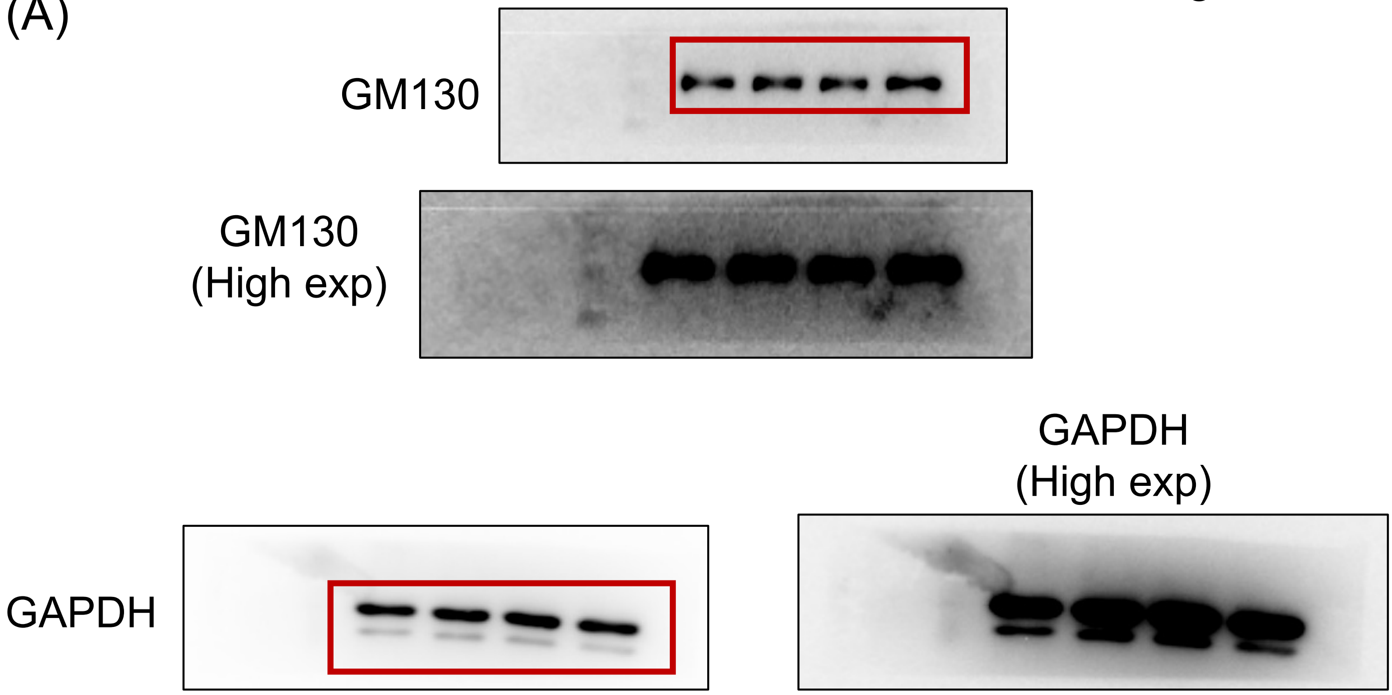

Biology Open • Supplementary information

Figure S4

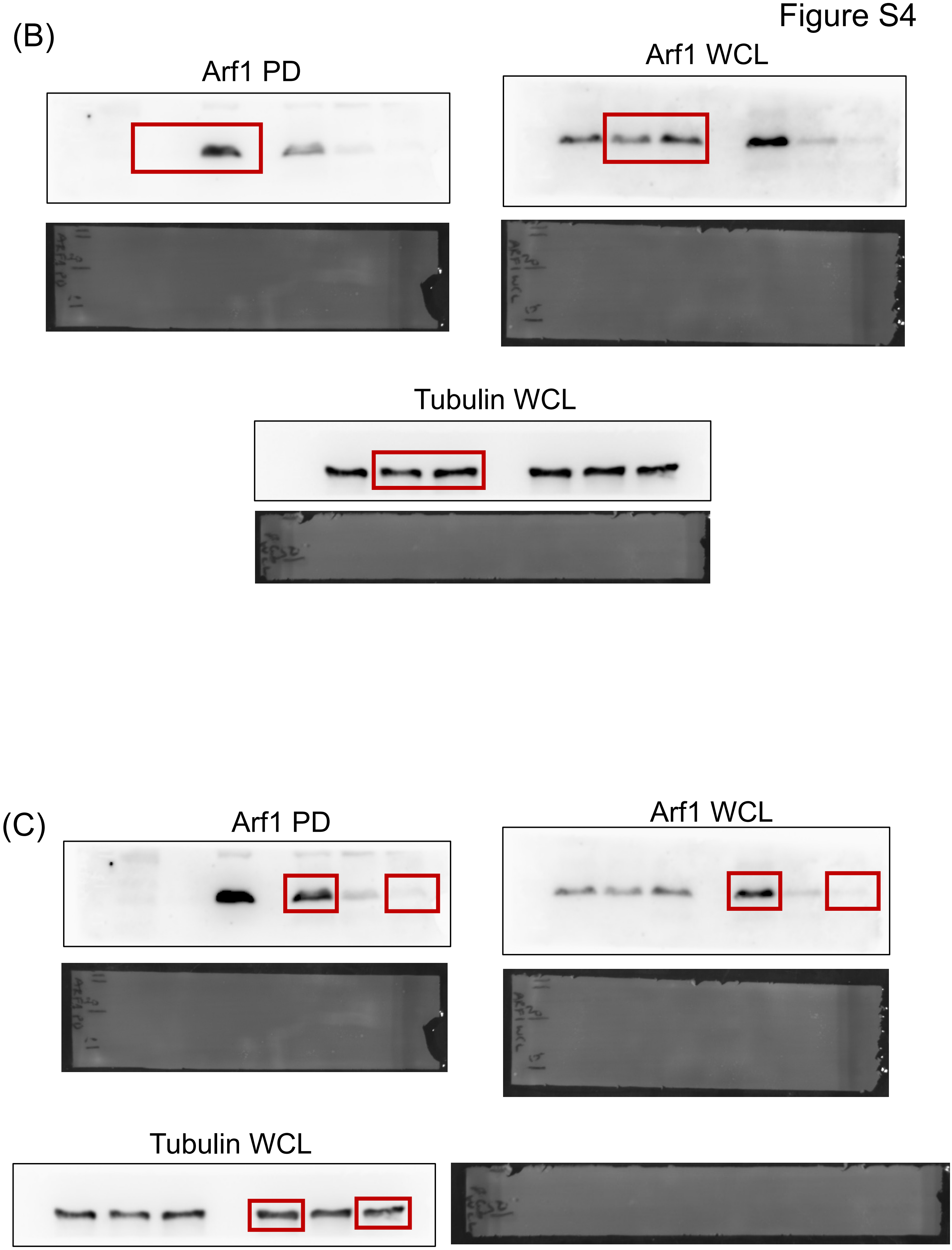

Biology Open • Supplementary information

Figure S4

(D)

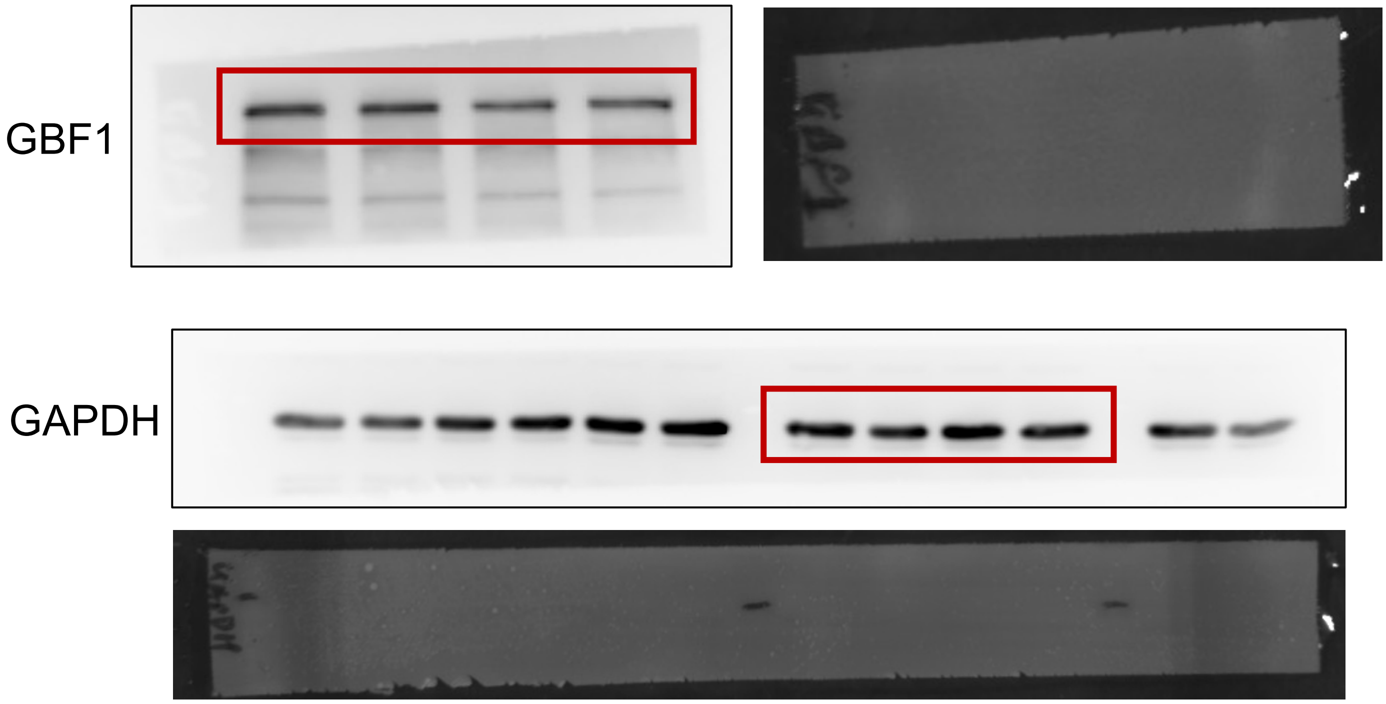

(F)

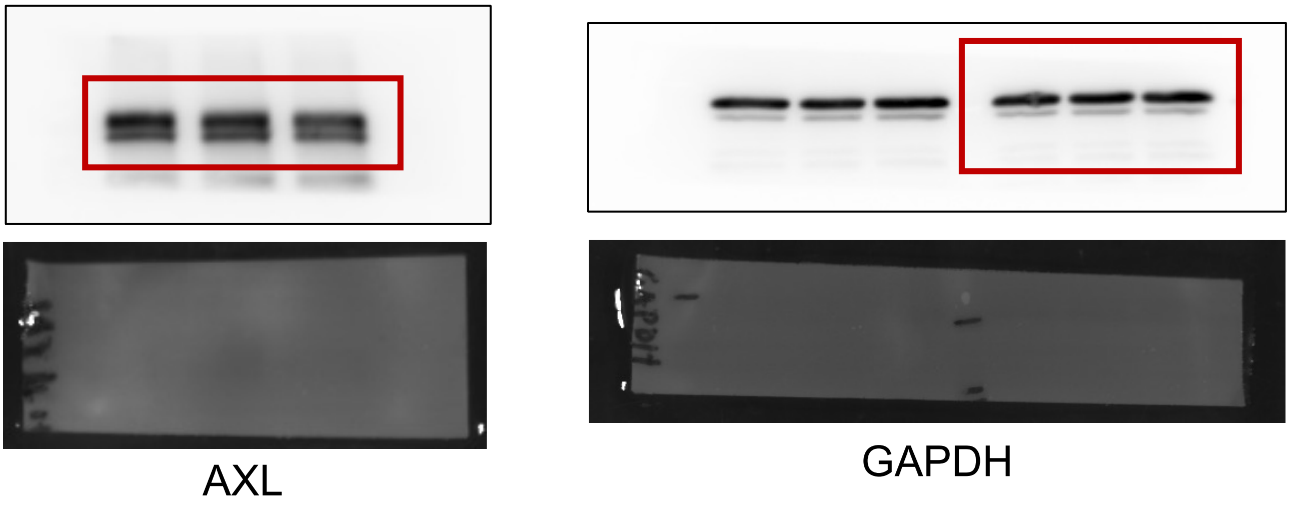

Biology Open • Supplementary information

Figure S4

(G)

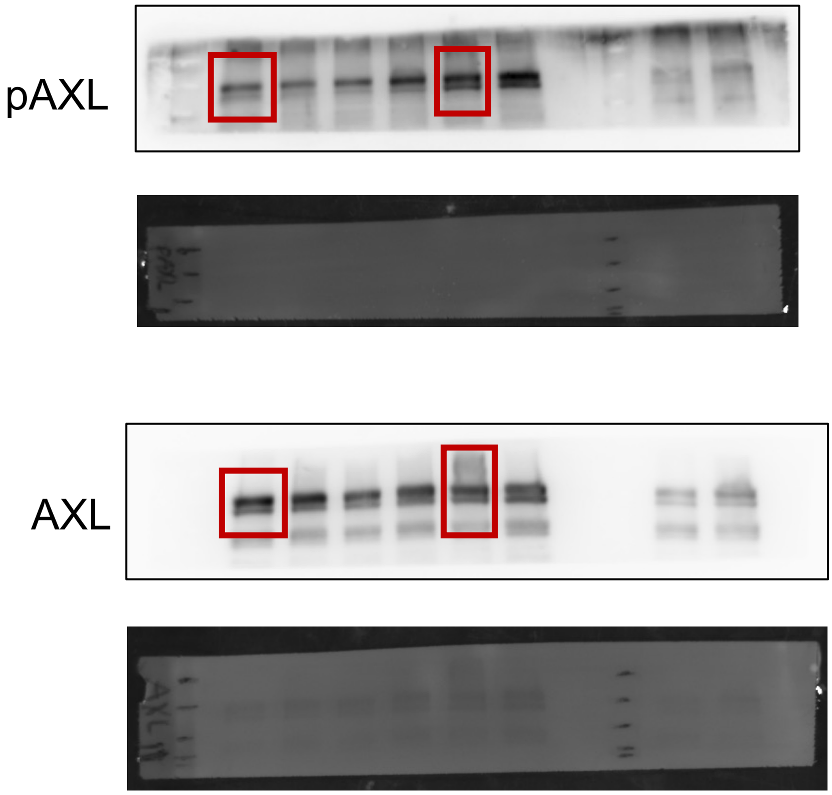

Figure S5

(A)

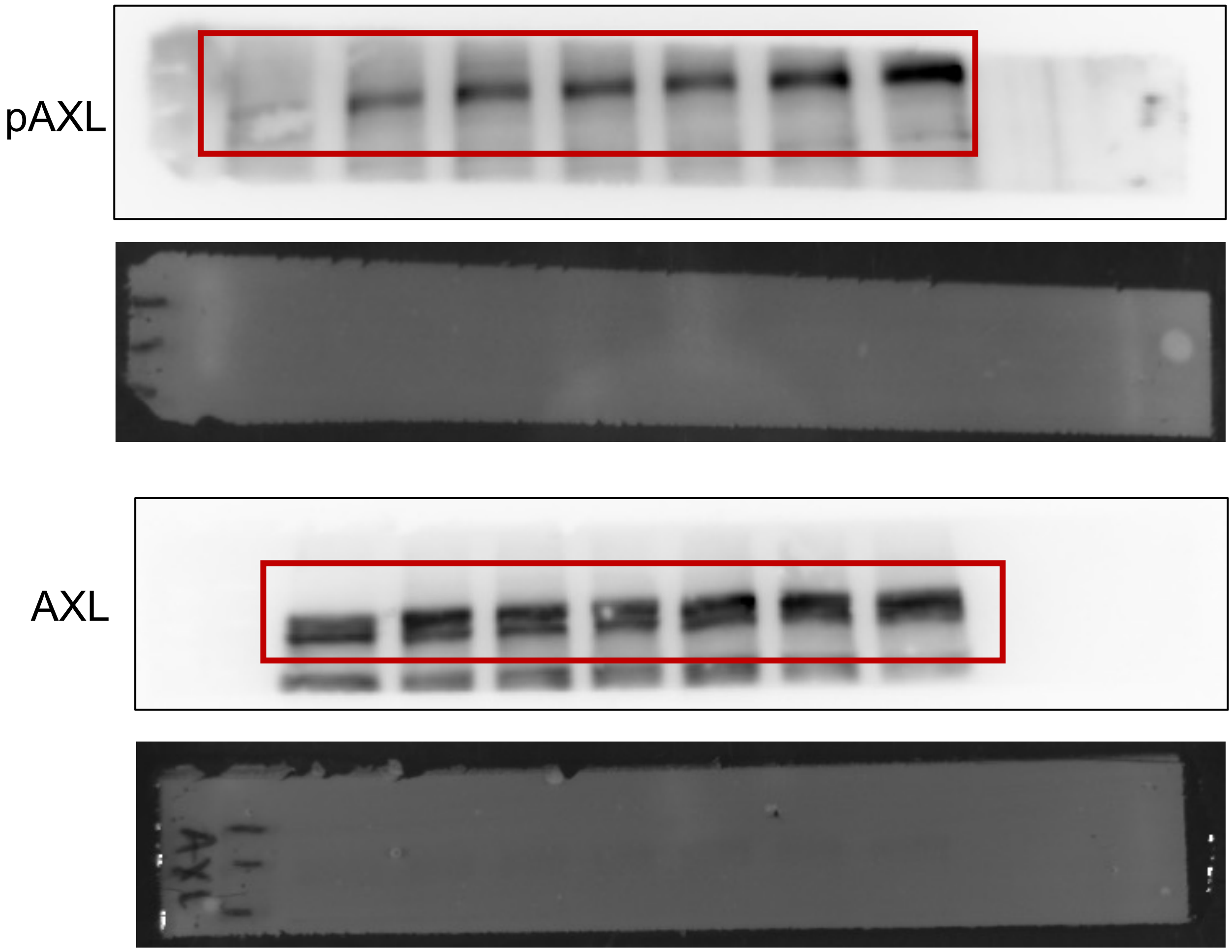

Biology Open • Supplementary information

Figure S5

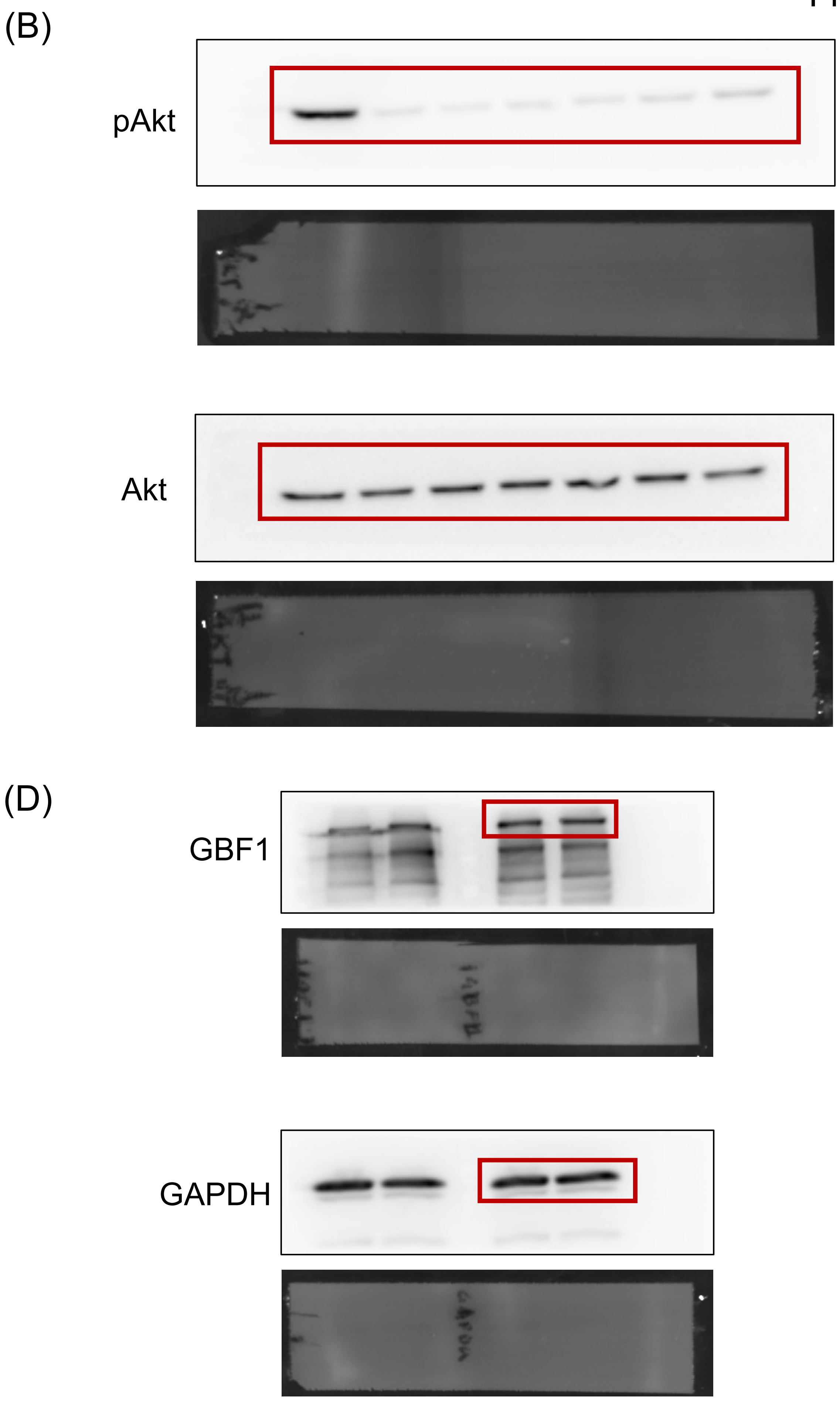

Fig. S7. Blot transparency data.

Biology Open • Supplementary information
